# Machine learning identifies ICU outcome predictors in a multicenter COVID-19 cohort

**DOI:** 10.1186/s13054-021-03720-4

**Published:** 2021-08-17

**Authors:** Harry Magunia, Simone Lederer, Raphael Verbuecheln, Bryant Joseph Gilot, Michael Koeppen, Helene A. Haeberle, Valbona Mirakaj, Pascal Hofmann, Gernot Marx, Johannes Bickenbach, Boris Nohe, Michael Lay, Claudia Spies, Andreas Edel, Fridtjof Schiefenhövel, Tim Rahmel, Christian Putensen, Timur Sellmann, Thea Koch, Timo Brandenburger, Detlef Kindgen-Milles, Thorsten Brenner, Marc Berger, Kai Zacharowski, Elisabeth Adam, Matthias Posch, Onnen Moerer, Christian S. Scheer, Daniel Sedding, Markus A. Weigand, Falk Fichtner, Carla Nau, Florian Prätsch, Thomas Wiesmann, Christian Koch, Gerhard Schneider, Tobias Lahmer, Andreas Straub, Andreas Meiser, Manfred Weiss, Bettina Jungwirth, Frank Wappler, Patrick Meybohm, Johannes Herrmann, Nisar Malek, Oliver Kohlbacher, Stephanie Biergans, Peter Rosenberger

**Affiliations:** 1 Department of Anesthesiology and Intensive Care Medicine, University Hospital Tübingen, Eberhard-Karls-University Tübingen, Hoppe Seyler Str. 3, 72076 Tübingen, Germany; 2 Institute for Translational Bioinformatics and Medical Data Integration Center, University Hospital Tübingen, Eberhard-Karls-University Tübingen, Tübingen, Germany; 3 Department of Intensive Care Medicine, University Hospital RWTH Aachen, Aachen, Germany; 4 Center for Anaesthesia, Intensive Care and Emergency Medicine, Zollernalb Klinikum, Balingen, Germany; 5 Department of Anesthesiology and Operative Intensive Care Medicine (CCM, CVK), Charité - Universitätsmedizin Berlin, Corporate Member of Freie Universität Berlin, Berlin Institute of Health, Humboldt-Universität zu Berlin, Berlin , Germany; 6 Department of Anesthesiology, Intensive Care Medicine/Pain Therapy, Knappschaftskrankenhaus Bochum, Bochum, Germany; 7 Department of Anaesthesiology and Intensive Care Medicine, University Hospital Bonn, Bonn, Germany; 8 Department of Anesthesiology and Intensive Care Medicine, Evangelisches Krankenhaus Bethesda, Duisburg, Germany; 9 Chair of Anesthesiology 1, Witten/Herdecke University, Wuppertal, Germany; 10 Department of Anesthesiology and Intensive Care Medicine, University Hospital Carl Gustav Carus, Technische Universität Dresden, Dresden, Germany; 11 Department of Anaesthesiology, University Hospital Düsseldorf, Düsseldorf, Germany; 12 Department of Anesthesiology and Intensive Care Medicine, University Hospital Essen, University Duisburg-Essen, Essen, Germany; 13 Department of Anaesthesiology, Intensive Care Medicine and Pain Therapy, University Hospital Frankfurt, Goethe University, Frankfurt, Germany; 14 Department of Anesthesiology and Critical Care, Medical Center - University of Freiburg, Freiburg, Germany; 15 Center for Anesthesiology, Emergency and Intensive Care Medicine, University of Göttingen, Göttingen, Germany; 16 Department of Anesthesiology, University Medicine Greifswald, Greifswald, Germany; 17 Department Cardiology, Angiology and Intensive Care Medicine, University Hospital Halle (Saale), Halle (Saale), Germany; 18 Department of Anesthesiology, Heidelberg University Hospital, Heidelberg, Germany; 19 Department of Anesthesiology and Intensive Care, Leipzig University Hospital, Leipzig, Germany; 20 Department of Anesthesiology and Intensive Care, University Medical Center Schleswig-Holstein, Campus Lübeck, University of Lübeck, Lübeck, Germany; 21 Department of Anaesthesiology and Intensive Care Therapy, Otto-Von-Guericke-University Magdeburg, Magdeburg, Germany; 22 University Hospital Marburg, UKGM, Philipps University Marburg, Marburg, Germany; 23 Department of Anesthesiology, Intensive Care Medicine and Pain Therapy, University Hospital Giessen and Marburg, Justus-Liebig University Giessen, Giessen, Germany; 24 Department of Anesthesiology and Intensive Care, School of Medicine, Klinikum Rechts Der Isar, Technical University of Munich, Munich, Germany; 25 Klinik Und Poliklinik Für Innere Medizin II, Klinikum Rechts Der Isar der, Technischen Universität München, Munich, Germany; 26 Department for Anesthesiology, Intensive Care Medicine, Emergency Medicine and Pain Medicine, St. Elisabethen Klinikum, Ravensburg, Germany; 27 Department of Anesthesiology, Intensive Care Medicine and Pain Medicine, Saarland University Hospital Medical Center, Homburg/Saar, Germany; 28 Department of Anesthesiology and Intensive Care Medicine, Ulm University, Ulm, Germany; 29 Department of Anaesthesiology and Intensive Care Medicine, Cologne-Merheim Medical Centre, Witten/Herdecke University, Cologne-Merheim, Germany; 30 Department of Anaesthesiology, Intensive Care, Emergency and Pain Medicine, University Hospital Wuerzburg, University Wuerzburg, Wuerzburg, Germany; 31 Department of Internal Medicine 1, University Hospital Tübingen, Tübingen, Germany; 32 Department of Computer Science, Institute for Bioinformatics and Medical Informatics, University of Tübingen, Tübingen, Germany; 33 Institute of Medical Informatics, Charité – Universitätsmedizin Berlin, Corporate Member of Freie Universität Berlin, Berlin Institute of Health, Humboldt-Universität Zu Berlin, Berlin, Germany

**Keywords:** COVID-19, Critical care, ARDS, Outcome, Prognostic models

## Abstract

**Background:**

Intensive Care Resources are heavily utilized during the COVID-19 pandemic. However, risk stratification and prediction of SARS-CoV-2 patient clinical outcomes upon ICU admission remain inadequate. This study aimed to develop a machine learning model, based on retrospective & prospective clinical data, to stratify patient risk and predict ICU survival and outcomes.

**Methods:**

A Germany-wide electronic registry was established to pseudonymously collect admission, therapeutic and discharge information of SARS-CoV-2 ICU patients retrospectively and prospectively. Machine learning approaches were evaluated for the accuracy and interpretability of predictions. The Explainable Boosting Machine approach was selected as the most suitable method. Individual, non-linear shape functions for predictive parameters and parameter interactions are reported.

**Results:**

1039 patients were included in the Explainable Boosting Machine model, 596 patients retrospectively collected, and 443 patients prospectively collected. The model for prediction of general ICU outcome was shown to be more reliable to predict “survival”. Age, inflammatory and thrombotic activity, and severity of ARDS at ICU admission were shown to be predictive of ICU survival. Patients’ age, pulmonary dysfunction and transfer from an external institution were predictors for ECMO therapy. The interaction of patient age with D-dimer levels on admission and creatinine levels with SOFA score without GCS were predictors for renal replacement therapy.

**Conclusions:**

Using Explainable Boosting Machine analysis, we confirmed and weighed previously reported and identified novel predictors for outcome in critically ill COVID-19 patients. Using this strategy, predictive modeling of COVID-19 ICU patient outcomes can be performed overcoming the limitations of linear regression models.

*Trial registration* “ClinicalTrials” (clinicaltrials.gov) under NCT04455451.

**Supplementary Information:**

The online version contains supplementary material available at 10.1186/s13054-021-03720-4.

## Background

The COVID-19 pandemic hit Germany in spring 2020 and since then intensive care resources were heavily utilized up to now [1]. Although large numbers of SARS-CoV-2 patients required intensive care unit (ICU) admission, ICU capacity in Germany was not exceeded. However, risk stratification and prediction of outcomes continues to be challenging. Several investigators have reported their ICU COVID-19 experience during this time period, yet these data show great variability in the number of cases and outcomes reported [2–16].

Few of these reports attempted to identify risk factors predicting morbidity, mortality and overall clinical outcome. This may be the result of the reporting of (1) incomplete data sets earlier in the pandemic as many patient were still undergoing ICU care for SARS-CoV-2 infection [10, 13, 15, 16], and/or (2) data sets biased by the need to triage ICU care to patients in the face of the exhaustion of local/regional ICU capacity [7, 10, 14, 15]. Nonetheless, there was consensus that SARS-CoV-2 ICU patients experienced lengthy ICU stays with ICU mortality in the range of 25 to 41% [14, 17]. Classical statistical analysis identified risk factors in these patient populations including age, renal function, the degree of pulmonary compromise and severity of acute respiratory distress syndrome (ARDS). But standard statistical techniques are limited in their ability to integrate diverse data types such as past medical history, therapeutic ICU interventions and many more in relation to clinical outcome variables [18].

To overcome these limitations, we employed machine learning methods to optimize risk stratification and prediction of overall outcomes for individual COVID-19 ICU patients. It has been recently shown that machine learning (ML) algorithms in combination with numerous, multidimensional variables with non-linear relationships may have advantages in clinical outcome prediction. Machine learning strategies were found to be superior to classical methods of outcome prediction typically used in cardiovascular pathologies [18, 19]. To take advantage of this superior technique for outcome prediction, we investigated 1186 PCR-confirmed COVID-19 patients receiving ICU care at 27 German hospitals that were enrolled retrospectively and prospectively. The aim of this study is to investigate whether ML can provide additional and interpretable insights for outcome prediction and weigh the identified outcome factors in COVID-19 ICU patients.

## Methods

### Study design, setting and participants

**Fig. 1:**
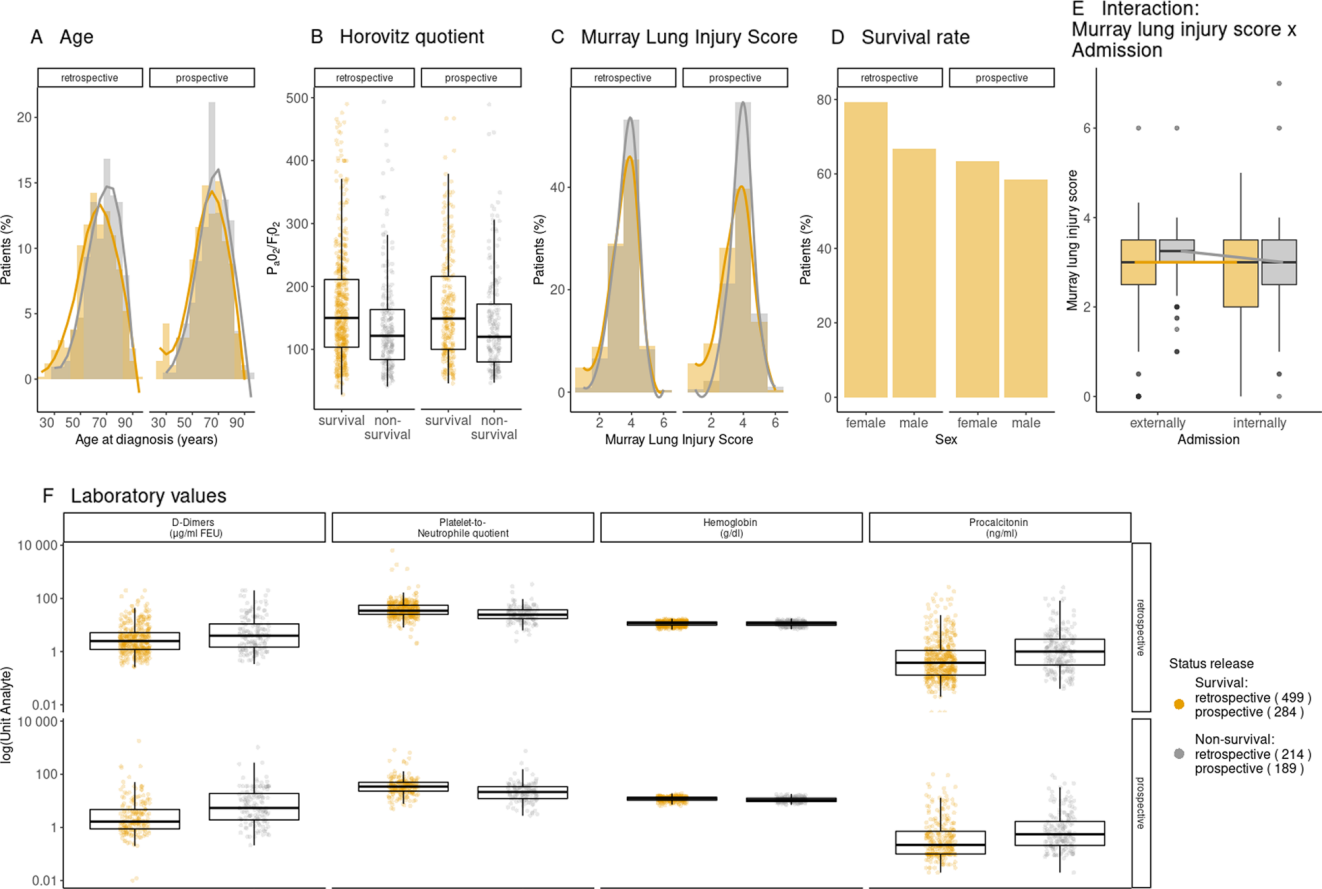
Descriptive data of patients included into the study population. (n = 596 retrospective cohort and n = 443 prospective cohort). **A** Distribution Age **B** Horovitz quotient at admission **C** Murray lung injury score and SOFA score without GCS at admission **D** Survival rates **E** Interaction of Murray long injury score and admission status **F** Laboratory values. Grey indicates patients that did not survive ICU therapy, orange indicates patients that did survive ICU therapy

This multi-center retrospective—prospective cohort study was performed with 27 participating German hospitals (Additional file 1: Table E1 and Fig. 1). An ethics approval was obtained from the participating hospitals’ Institutional Review Boards. The study was registered in “ClinicalTrials” (clinicaltrials.gov) under NCT04455451. COVID-19 patients 18 years and older requiring ICU admission between 1st January 2020 and 4th May 2021 at a participating center were recruited for this study. Patients were recruited either retrospectively (1st January 2020 to 31st July 2020) or prospectively (29th September 2020 to 4th May 2021). Inclusion criteria were the requirement for ICU treatment due to COVID-19 confirmed by a positive SARS-CoV-2 PCR test. The local investigator confirmed the accuracy and completeness of all entered data. A secure electronic research data capture system (REDCap) was used to collect and manage study data in a pseudonymous fashion [20, 21].

**Table 1:** Clinical characteristics of N = 1186 patients included in the study; clinical and laboratory parameters

Parameter	Total N	Missing N	Non-survival	Survival	Total
Total N (%)			403 (34.0)	783 (66.0)	1186
Age (years)	1186	0	66.0 (58.0 to 75.5)	62.0 (53.0 to 72.0)	63.0 (54.0 to 73.0)
*Age groups*	1186	0			
18–29 years			2 (0.5)	26 (3.3)	28 (2.4)
30–39 years			8 (2.0)	37 (4.7)	45 (3.8)
40–49 years			32 (7.9)	75 (9.6)	107 (9.0)
50–59 years			75 (18.6)	196 (25.0)	271 (22.8)
60–69 years			130 (32.3)	202 (25.8)	332 (28.0)
70–79 years			105 (26.1)	187 (23.9)	292 (24.6)
80–89 years			48 (11.9)	57 (7.3)	105 (8.9)
> 90 years			3 (0.7)	3 (0.4)	6 (0.5)
*Sex*	1186	0			
Female			92 (22.8)	241 (30.8)	333 (28.1)
Male			311 (77.2)	542 (69.2)	853 (71.9)
BMI (kg/m^2^)	1120	66	28.1 (25.1 to 33.1)	28.4 (25.2 to 32.7)	28.3 (25.2 to 32.8)
*BMI groups*	1120	66			
Below 20 kg/m^2^			8 (2.1)	16 (2.2)	24 (2.1)
20–25 kg/m^2^			88 (22.8)	164 (22.3)	252 (22.5)
25–30 kg/m^2^			141 (36.5)	267 (36.4)	408 (36.4)
Above 30 kg/m^2^			149 (38.6)	287 (39.1)	436 (38.9)
*Bloodgroup*	755	431			
0			121 (40.2)	158 (34.8)	279 (37.0)
A			124 (41.2)	213 (46.9)	337 (44.6)
AB			10 (3.3)	24 (5.3)	34 (4.5)
B			46 (15.3)	59 (13.0)	105 (13.9)
*Past medical history and chronic medications*					
Arterial hypertension	1186	0	255 (63.3)	479 (61.2)	734 (61.9)
Cardiovascular disease	1186	0	124 (30.8)	187 (23.9)	311 (26.2)
Chronic arrhythmia	1186	0	61 (15.1)	84 (10.7)	145 (12.2)
COPD	1186	0	38 (9.4)	69 (8.8)	107 (9.0)
Other lung disease	1186	0	44 (10.9)	80 (10.2)	124 (10.5)
Nicotine abuse	1186	0	36 (8.9)	78 (10.0)	114 (9.6)
History of solid organ transplant	1186	0	9 (2.2)	14 (1.8)	23 (1.9)
History of bone marrow transplant	1186	0	3 (0.7)	5 (0.6)	8 (0.7)
Alcoholism	1186	0	13 (3.2)	23 (2.9)	36 (3.0)
Chronic kidney failure	1186	0	53 (13.2)	92 (11.7)	145 (12.2)
Diabetes mellitus	1186	0	126 (31.3)	218 (27.8)	344 (29.0)
NIDDM	1186	0	88 (21.8)	132 (16.9)	220 (18.5)
Prior thrombotic events^a^	1186	0	24 (6.0)	35 (4.5)	59 (5.0)
ACE inhibitors	1186	0	95 (23.6)	172 (22.0)	267 (22.5)
AT2 receptor blocker	1186	0	47 (11.7)	117 (14.9)	164 (13.8)
Beta blockers	1186	0	116 (28.8)	227 (29.0)	343 (28.9)
Anti-platelet medication	1186	0	95 (23.6)	165 (21.1)	260 (21.9)
NOAC	1186	0	29 (7.2)	56 (7.2)	85 (7.2)
Corticosteroids	1186	0	44 (10.9)	63 (8.0)	107 (9.0)
Immunosuppressive drugs	1186	0	21 (5.2)	31 (4.0)	52 (4.4)
Opioids	1186	0	19 (4.7)	38 (4.9)	57 (4.8)
*Status at ICU admission*					
Admission/Transfer status	1186	0			
External transfer			206 (51.1)	336 (42.9)	542 (45.7)
Internal or direct admission			197 (48.9)	447 (57.1)	644 (54.3)
Ventilatory status at admission	1186	0			
Intubated			217 (53.8)	278 (35.5)	495 (41.7)
Non-invasive assisted ventilation			40 (9.9)	91 (11.6)	131 (11.0)
Spontaneous breathing			146 (36.2)	414 (52.9)	560 (47.2)
Prior days of non-invasive ventilation	1016	170	0.0 (0.0 to 1.0)	0.0 (0.0 to 0.0)	0.0 (0.0 to 0.0)
Days prior invasive ventilation	1099	87	0.0 (0.0 to 3.0)	0.0 (0.0 to 1.0)	0.0 (0.0 to 1.0)
RASS	1100	86	-2 (-5 to 0)	0 (-4 to 0)	0 (-4 to 0)
SOFA (w/o GCS)	1186	0	7 (4 to 9)	5 (3 to 7)	5 (3 to 8)
Murray Lung Injury Score	1156	30	3.2 (2.5 to 3.5)	3.0 (2.2 to 3.5)	3.0 (2.5 to 3.5)
ARDS grading according to PaO2/FiO2 quotient	1154	32			
Mild (PaO2/FiO2 201 to 300)			45 (11.3)	147 (19.4)	192 (16.6)
Moderate (PaO2/FiO2 101 to 200)			189 (47.6)	357 (47.2)	546 (47.3)
Severe (PaO2/FiO2 < = 100)			142 (35.8)	186 (24.6)	328 (28.4)
No ARDS			21 (5.3)	67 (8.9)	88 (7.6)
Static compliance (ml/mbar)^b^	612	574	34.6 (24.7 to 44.4)	37.9 (29.3 to 49.4)	36.7 (27.5 to 47.3)
Driving pressure (mbar)^b^	636	550	13.0 (10.0 to 16.0)	12.0 (10.0 to 15.0)	12.0 (10.0 to 15.0)
Hemoglobin (g/dl)	1179	7	11.0 (9.5 to 12.9)	11.9 (10.1 to 13.3)	11.6 (9.9 to 13.2)
Platelets (x10^3^µl-1)	1178	8	203.0 (146.0 to 282.0)	232.0 (177.0 to 316.0)	223.0 (164.0 to 304.0)
Leucocytes (n/nl)	1177	9	10.7 (7.1 to 14.5)	8.8 (6.1 to 12.1)	9.4 (6.3 to 12.9)
Lymphocytes (n/nl)	971	215	0.7 (0.4 to 1.1)	0.8 (0.6 to 1.2)	0.8 (0.5 to 1.2)
Neutrophiles (n/nl)	886	300	8.7 (5.6 to 12.7)	6.8 (4.7 to 9.4)	7.3 (4.9 to 10.5)
Platelet/neutrophile ratio	882	304	23.3 (15.5 to 36.0)	34.6 (24.9 to 53.2)	31.4 (21.2 to 47.5)
Platelet/lymphocyte ratio	965	221	278.1 (158.3 to 473.9)	280.4 (181.8 to 425.7)	280.3 (174.1 to 436.6)
C-reactive protein (mg/dl)	1141	45	17.5 (9.6 to 27.9)	14.1 (7.6 to 22.5)	14.8 (8.4 to 24.3)
Procalcitonin (ng/ml)	1153	33	0.7 (0.3 to 2.4)	0.3 (0.1 to 0.9)	0.4 (0.1 to 1.4)
Interleukin-6 (pg/ml)	851	335	161.0 (63.0 to 429.5)	89.4 (39.8 to 189.5)	104.0 (43.2 to 268.9)
Ferritin (µg/dl)	623	563	154.0 (88.3 to 272.8)	114.2 (59.2 to 195.5)	126.3 (68.8 to 210.8)
D-Dimer (µg/ml)	905	281	4.2 (1.7 to 14.9)	2.2 (1.1 to 5.0)	2.8 (1.2 to 8.0)
Total bilirubin (mg/dl)	1160	26	0.7 (0.4 to 1.1)	0.6 (0.4 to 0.8)	0.6 (0.4 to 0.9)
Creatinine (mg/dl)	1172	14	1.2 (0.8 to 2.1)	0.9 (0.7 to 1.4)	1.0 (0.8 to 1.6)
*ICU outcomes*					
Mortality n/%			403 (34.0)		
LOS ICU (days)	1186	0	14.0 (8.0 to 24.0)	16.0 (6.0 to 34.0)	15.0 (7.0 to 30.0)
Transfer destination	1180	6			
Intermediate care			n/a	51 (6.6)	n/a
Normal ward			n/a	489 (62.9)	n/a
Other ICU			n/a	125 (16.1)	n/a
REHAB			n/a	112 (14.4)	n/a

All values are reported as absolute numbers and percentages for categorical variables, and as median and interquartile ranges (IQR) if not distributed normally for continuous variables

BMI, body-mass-index (kg/m^2^); COPD, chronic-obstructive pulmonary disease; ICU, intensive care unit; LOS, length-of-stay; NIDDM, Non-insulin dependent diabetes mellitus; NOAC, novel oral anticoagulants; RASS, Richmond agitation sedation scale; SOFA score without GCS, sequential organ failure assessment score without Glasgow coma scale (GCS); n/a not available

^a^Prior thrombotic events: e.g. deep vein thrombosis, pulmonary embolism

^b^At ICU admission or first value after intubation at day of ICU admission

### Variables and measurements

During the data collection process demographic data, past medical history, previous medications, current illness data, laboratory values as well as outcome data were collected. A total of 49 variables were used for the ML models (Additional file 1: Table E8).

To allow comparability of intubated and spontaneously breathing patients the Sequential Organ Failure Assessment (SOFA) score was calculated without the Glasgow Coma Scale (GCS) [22]. Murray Lung injury score was calculated as previously published [23]. Static compliance and driving pressure were calculated as previously described [24]. Laboratory values were converted to a common unit to permit analysis. Oxygen supply in spontaneously breathing patients was converted to an estimated F_i_O_2_ (Additional file 1: Table E2).

**Table 2:** Overall performance of the machine learning models for ICU outcome prediction

Prediction variable	ICU survival N = 1053		ECMO therapy N = 1053		Renal Replacement Therapy N = 1000	
	Balanced accuracy	PR-AUC	Balanced accuracy	PR-AUC	Balanced accuracy	PR-AUC
*ML model*						
RF	0.65	0.84	0.72	0.75	0.69	0.66
SVC	0.65	0.81	0.79	0.53	0.7	0.64
EBM	0.61		0.72		0.68	
EBM (10 interactions)	0.64	0.81	0.73	0.69	0.7	0.69

EBM, explainable boosting machine; ICU, intensive care unit; ML, machine learning; RF, random forest classifier; SVC, support vector classifier

### Bias management

#### Discontinuation of ICU care

107 (8.3%) patients, or their legal representative requested that ICU level care be discontinued during the ICU stay. The majority of these patients died during the ICU stay (n = 95, 88.8%). To avoid bias in predictor analyses this patient group was excluded from further analyses (for patient characteristics please see Additional file 1: Table E5). For three patients these data were not available, they were excluded from the analyses.

#### Dataset used for ML

Out of the 1186 patients included in the final study (Table 1), 147 were transferred to another ICU. Due to study design the ultimate ICU outcome of this subset of patients is unknown. To avoid bias in survival prediction these patients were excluded, thus ML models were trained on 1039 (complete cohort), 596 (retrospective cohort) and 443 (prospective cohort) patients.

### Statistical analyses

Observed parameters were assessed for their distribution. Outliers were excluded by visual assessment of clinical validity based on the distribution plots (excluded data points are provided in Additional file 1: Table E3). Baseline characteristics of all patients were evaluated. Continuous variables are reported as either means and standard deviation (SD) if normally distributed or as median and interquartile ranges (IQR) if not normally distributed. Shapiro–Wilk-Test was used prior to Student’s t test or Wilcoxon rank sum test. Kaplan–Meier estimators were compared using Log-Rank-Test. Categorical variables were compared using the Fisher’s Exact Test. A sample size calculation was not performed. Study size is defined by the available datasets in the recruitment period. All statistical analyses were performed in R (version 4.0.3) and JMP (version 15.2.0, SAS Institute, Cary, USA).

### Description of machine learning process

Variables are referred to as features in machine learning (ML) but for consistency we will refer to them as variables. For a detailed description of the machine learning process please see Additional file 1: Table E7. We trained Support Vector Classifier (SVC), Random Forest Classifier (RF), and EBM with a fivefold stratified Cross Validation (CV) by using 80% of the data for training and 20% of the data for testing. We excluded variables with more than 30% of data missing (see Additional file 1: Table E7). For all ML-methods, we applied one-hot encoding for categorical data, i.e. creating indicator columns for each category (including missing values). We converted Boolean data to numerical values zero and one. We performed a hyper-parameter optimization across all ML-algorithms with nested CV techniques [25]. Performance of the models was evaluated as the average of balanced accuracy and the area under precision-recall curve (PR-AUC) per fold of CV. A regular accuracy or AUC would be biased towards the overrepresented class (“survival”). In order to verify the robustness of our results in light of the imbalanced outcome variable, we used both over-sampling and under-sampling for the outcome “survival”. For over-sampling, the observations from the under-represented class (here: “non-survival”) were added at random to the data set. For under-sampling, the over-represented class (here”survival”) was reduced at random to the same size as the underrepresented class. We compared the ranking of variable importance and the shape function with the results from each of the fivefold stratified CV runs on the retrospective dataset. The results of each run were the same (data not shown). We further validated the results by training the ML-models with a fivefold CV for hyper-parameter optimization (RF and SVC) on the retrospective data and predicting the outcome on the prospective data (see Table 2).

For the results presented in this paper, we trained the EBM on the entire dataset (retrospective and prospective).

#### Rationale for the use of the explainable boosting machines model

**Fig. 2:**
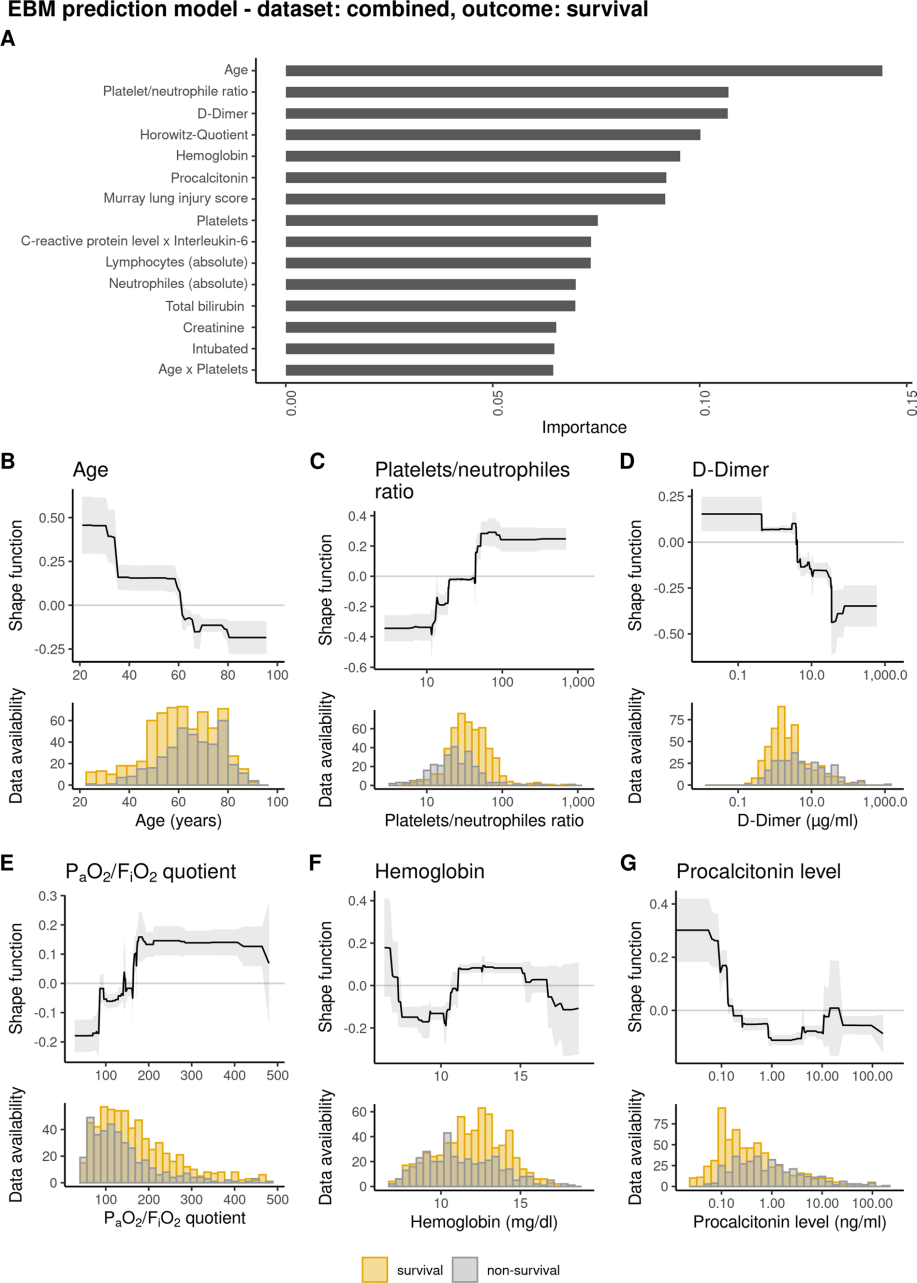
EBM prediction model showing importance of risk factors predicting “survival” in COVID-19 ICU patients including admission data. Top **A** significant risk factors for outcome after analysis of admission data and weighed according to their importance for outcome. bottom) **B** importance of age for outcome and distribution of age data **C** platelet/neutrophil ratio and distribution of data on admission **D** initial D-dimer serum values and distribution of data determined on admission **E** importance of Horovitz quotient (P_a_O_2_/F_i_O_2_) for outcome and distribution of data on admission **F** initial hemoglobin values and distribution of data on admission **G** initial procalcitonin (PCT) serum values and distribution of data on admission. Grey indicates patients that did not survive ICU therapy, orange indicates patients that did survive ICU therapy

EBMs are built on a generalized additive model (GAM) of the form

$$g\left( y \right) = \varpi_{1} f_{1} \left( {x_{1} } \right) + \varpi_{2} f_{2} \left( {x_{2} } \right) + ::: + \varpi_{p} f_{p} \left( {x_{p} } \right),$$

where $g$ is the link function and $f_{i} \left( {x_{i} } \right)$ the shape function for variable $x_{i}$ and $\varpi$_i_ is the weight for variable $x_{1}$, with which each variable influences the model. In a classification problem, the link function $g$ is a logistic function [26]. As the model is additive, each variable contributes in a modular way. This allows for an easy interpretation about the influence of a variable to the prediction (see Fig. 2A). The idea of using shape functions for each variable allows for complex relationships (even non-linear) between the variable and the outcome prediction (see Fig. 2B). Therefore, GAMs can be significantly more accurate than simple linear models [27]. We use EBMs as they additionally employ modern machine learning techniques such as bagging and boosting and have a comparable performance to state-of-the art ML techniques such as RF [27, 28]. Overall performance of the ML models was assessed by balanced accuracy and PR-AUC (Table 2).

## Results

### Participating centers and level of care

27 ICUs participated in this observational study including 24 ICUs from university hospitals and three ICUs from regional primary and secondary care hospitals (Additional file 1: Table E1, Figure E1). All patients requiring ICU treatment could receive the full treatment possibilities including ventilation, renal replacement therapy (RRT), and extracorporeal membrane oxygenation (ECMO).

### Patient characteristics and status at ICU admission

1186 patients were recruited into the study (patient selection chart, Additional file 1: Figure E2) with 713 patients in the retrospective and 473 patients in the prospective cohort. Overall patient characteristics, severity of the disease, and organ failure are given in Table 1 and Additional file 1: Table E4. Twice as many males (71.9%) than females (28.1%) were treated at the participating ICUs. The median age was 63 (IQR 54 to 73), 180 patients (15.2%) had an age below 50 years, and 6 patients (0.5%) had an age above 90 years. For age distribution and baseline parameters please see Fig. 1. Kaplan Meier Curves for probability of ICU survival according to patient age are provided in Additional file 1: Figure E3a. At ICU admission spontaneous breathing via oxygen mask, non-invasive assisted ventilation or invasive ventilation were present in 47.2%, 11%, 41.7% patients, respectively. Data for the grading of the ARDS severity were available for 1154 patients (97.3%). According to the Berlin definition ARDS was graded using the P_a_O_2_/F_i_O_2_ index as mild (16.6%), moderate (47.3%), or severe (28.4%) [29]. Additional file 1: Figure E3b provides the Kaplan Meier Curves for probability of ICU survival according to ARDS severity.

**Fig. 3:**
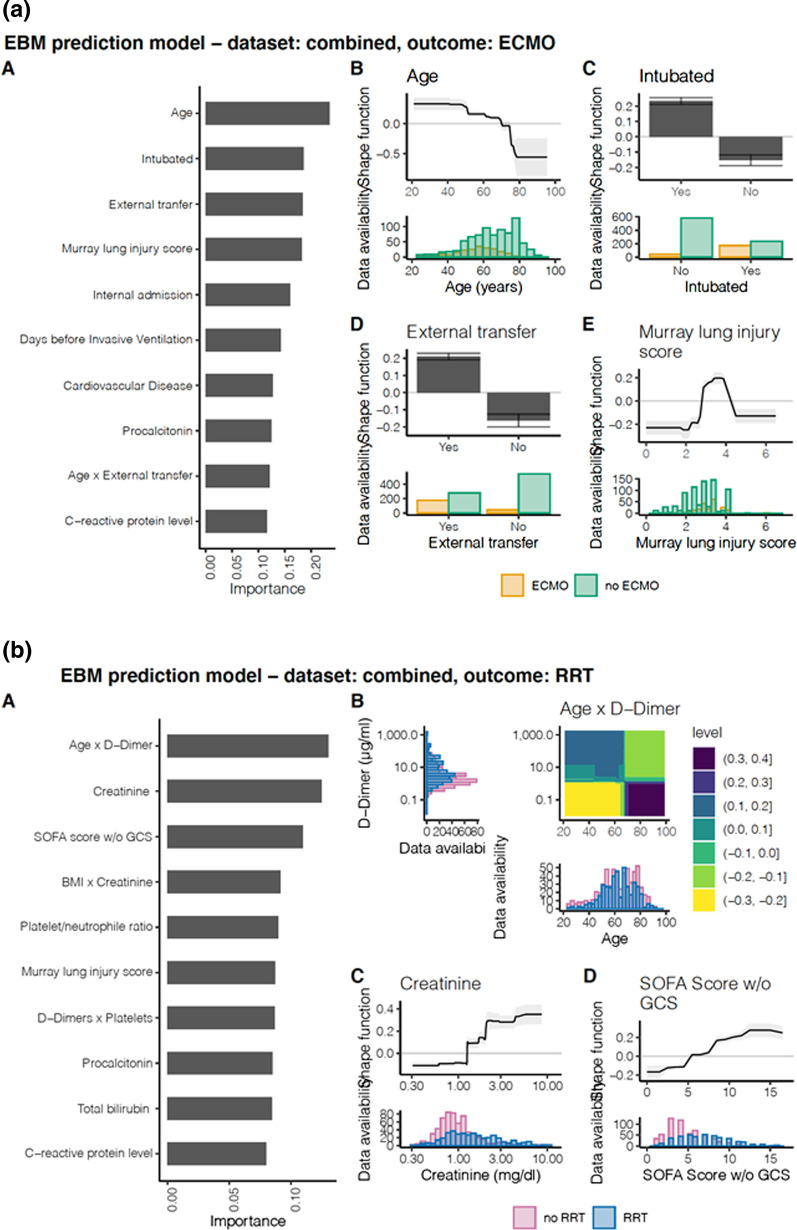
EBM prediction model showing importance of risk factors predicting need for ECMO or RRT in COVID-19 ICU patients including admission data. (a) ECMO therapy left) **A** significant risk factors for outcome after analysis of admission data and weighed according to their importance for outcome. Right **B** importance of age for outcome and distribution of age data **C** importance of status “intubated” on ICU admission and distribution of status D) importance of status “external transfer” on ICU admission and distribution of status **E** importance of Murray lung injury score and distribution of MLIS data. Green indicates patients that did not receive ECMO therapy, orange indicates patients that did receive ECMO therapy. (b) Renal Replacement Therapy (RRT). Left **A** significant risk factors for outcome after analysis of admission data and weighed according to their importance for outcome. Right **B** importance of the interaction of age and D-dimer level for outcome and distribution of data **C** initial creatinine values and distribution of data determined on admission **D** initial SOFA score w/o GCS and distribution of data determined on ICU admission. Blue indicates patients that did not receive RRT, red indicates patients that did receive RRT

### Patient outcome

Overall ICU mortality was 34% for all recruited patients. Median length of ICU stay was 15 days (IQR 7 to 30 days). Mortality was significantly lower in female patients (27.6%) than in male patients (36.5%) (*p* = 0.0041). Mortality was highest in octogenarians with an observed mortality of 45.7% (Additional file 1: Figure E3a). 22% patients received ECMO therapy (21% in the retrospective cohort and 23.5% in the prospective cohort) with a median duration of 16 days (IQR 9 to 26). 95% of patients received veno-venous ECMO, 2% of patients received a veno-arterial ECMO and 3% received a transition from veno-venous to veno-arterial ECMO. Patients receiving ECMO therapy were significantly younger than those not receiving ECMO (57 (IQR 49 to 65) years vs. 66 (IQR 56 to 76) years; *p* < 0.0001). 39.3% patients, not receiving chronic dialysis prior to ICU admission, received RRT/dialysis therapy during their ICU stay (41.7% in the retrospective cohort and 35.8% in the prospective cohort).

### Prediction of ICU survival by EBM models

Overall performance of the different ML models including results for balanced accuracies and precision recall area under the curve (PR-AUC) are given in Table 2. The EBM model based on variables reflecting status at ICU admission (Additional file 1: Table E8), resulted in a high precision recall area under the curve (PR-AUC) of 0.81 and a moderate balanced accuracy of 0.64 (Additional file 1: Figure E4a). The ten most important predictive variables in the admission model were according to their predictive importance: age, platelet/neutrophil ratio, D-dimer, Horowitz quotient, hemoglobin, procalcitonin, Murray lung injury score, platelet count, interaction of c-reactive protein and interleukin-6 and absolute lymphocyte count (Fig. 2). Patients’ comorbidities were not under the fifteen most important variables. As shown in the shape function for the variable age, there is a transition from improved survival to worsened survival at the age of 61 years (confidence interval (CI) 60 to 62) with a first worsening at the age of 34.7 (CI 31 to 35) years. The platelet/neutrophil ratio was the second most important parameter showing a worsened outcome above a ratio of 43.7 (CI 19.6 to 44.1). Elevated D-Dimers, for instance, affect ICU survival negatively at levels above 4.06 µg/ml (CI 3.78 to 4.07). Low Horovitz quotients demonstrated a negative impact on ICU survival with transitions for the worst impact at P_a_O_2_/F_i_O_2_ quotients below 85 (CI 84 to 86) and improved survival above 163 to 172. Overall performance and results of the EBM model was similar for the different datasets (complete, prospective and retrospective) (Additional file 1: Table E9, Figure E5a).

### Predicting the need for ECMO therapy by EBM models

EBM models for the prediction of ECMO therapy resulted in a good PR-AUC of 0.69 and a good balanced accuracy of 0.73. The five most important parameters associated with ECMO therapy according to their predictive importance were: age, ventilatory status “intubated” at ICU admission, admission by external transfer, Murray lung injury score, and admission by internal transfer (reduced risk) (Fig. 3a). The shape function for the factor age showed a higher risk for ECMO therapy below the age of 70 (CI 69 to 75) years. A Murray Lung injury score above a level of 2.8 (no CI) resulted in a higher risk for ECMO therapy. Patients admitted by external transfer had a higher risk to receive ECMO therapy. Comparison of the EBM models and selected shape functions of important variables revealed similar results (Additional file 1: Table E9 and Figure E5b).

### Prediction of renal replacement therapy by EBM models

Patients on chronic dialysis were excluded prior to EBM model generation. The EBM model on the complete dataset resulted in a good PR-AUC (Additional file 1: Figure E4c). The five most important parameters according to their predictive importance were: interaction of age with D-dimer level, creatinine level, SOFA score w/o GCS, interaction of BMI with creatinine, and platelet/neutrophile ratio (Fig. 3b). Patients with an age below approximately 65 years combined with elevated D-dimers had a higher risk for the need of RRT (see heatmap of interaction of age and D-dimers in Fig. 3b). An elevated creatinine level above 1.3 mg/dl (no CI) at ICU admission, as well as a SOFA score w/o GCS above 5 (no CI) resulted in a higher risk to receive RRT during ICU stay. Throughout all EBM models, creatinine and bilirubin levels showed a reverse correlation relationship.

## Discussion

In this multi-center retrospective—prospective cohort study we identified and weighed possible predictive factors on COVID-19 outcome using a machine learning approach on 49 variables. Using the present ML approach, we confirmed previously reported factors and extend knowledge to novel factors and factor combinations likely predicting outcome in COVID-19 patients. Shape functions for each of these variables show the individual influence of the variable for the prediction of the outcome. For ICU survival these include age, platelet/neutrophil ratio, D-dimers, and ARDS severity. The most important factors for the prediction of RRT need include the combination of Age and D-Dimers, Creatinine levels and SOFA score without GCS.

Previous studies have shown that older age, obesity, diabetes, being immunocompromised, lower P_a_O_2_/F_i_O_2_, higher hemodynamic and renal SOFA score at ICU admission were independently associated with 90-day mortality in COVID-19 [14]. This has also been reported by other investigators, yet they did not show individual cutoff values nor weigh the individual importance for the identified factors [30, 31]. To exclude an early effect or a late effect as seen when logistic regression is performed, we included almost all admission variables collected for our cohort. Variable selection influencing outcome can be performed in ML models but is less crucial than for logistic regression. We refrained from such a variable selection in our EBM model’s decision process. In our analysis we were able to confirm that age and pulmonary function on admission are important predictors in COVID-19 ICU patients. The present shape functions clearly show a non-linear association between the predictive factors and the outcome variable. Patient’s age, for instance, as the most important predictive factor, shows a higher chance for ICU survival below 61 years. Additionally, the ML approach identified the D-dimer level and platelet/neutrophil ratio at ICU admission as important factors. This is especially interesting in the context of reported thrombotic complications of COVID-19 patients [32, 33]. When activated, neutrophils complex with platelets to form platelet-neutrophil complexes (PNCs) activating both cell types. These PNCs enhance inflammation, increases neutrophil extracellular trap formation, and result in micro-thrombosis [34, 35]. The same is applicable when looking at D-dimer levels. High D-dimer levels reflect an activation of inflammation and the formation of micro-thrombi with neutrophil extracellular trap formation. We can therefore say that our data reflects the inflammatory markers known from translational science and confirm their relevance to outcome [35].

In everyday clinical practice, it is of great interest to assess the further course of patients in intensive care, such as a necessity for renal replacement or ECMO therapy. The present ML model predicting the need for ECMO therapy identified age and pulmonary compromise (Murray lung injury score) as important factors. Admission both from an external hospital and already in an intubated state are associated with the need for ECMO therapy. This result is not surprising, as both younger and more severely pulmonary compromised patients were typically transferred for ECMO therapy to our participating centers [36]. Our ML models assessing the need for RRT include age as an important factor as well as variables quantifying disease severity (SOFA score) or inflammatory and thrombotic activity (D-dimers and Platelet/neutrophil ratio). Our models do not only permit the identification of risk factors in COVID-19 patients, they also provide insights to the weight of each individual variable for the selected ICU outcome of the individual patient [18, 37]. The ML models chosen allow for transparent assessment of various variables in a non-linear fashion which overcomes limitations of currently employed regression models. The use of shape functions in GAMs for each variable allows for complex relationships (even non-linear) between the variable and the outcome prediction. Therefore, EBMs can be significantly more accurate than simple linear models [27]. Interactions of different variables extend the analyzing capabilities of the ML approach. Overall, the results from the EBM offer a greater degree of interpretability than a p-value of a linear regression, or an odds ratio analysis. As shown in Figs. 2 and 3 the visualizations offer insight into transition values from positive to negative impact, plateaus, as well as confidence intervals as a certainty measure.

A limitation of the present study is that we were not able to include even more patients into the analysis. This is of course a valid point of criticism, yet the data used for our analyses were manually collected and curated. The data was not simply exported from an electronic medical record where missing data are prevalent and validity of the information has not been confirmed. Missing data often needs to be imputed prior to analysis. As a result of the design of our study, we were largely able to reduce imputation of missing data, again adding to the significance of our findings. The predictiveness of the models presented here differed for the three outcomes (survival, ECMO, RRT). This is likely due to the underlying dataset containing more information for predicting e.g. survival compared to ECMO. Since the study was designed with a focus on predicting survival, some variables which might better predict ECMO or RRT might not have been included in this study (for details see Additional file 1: Table E9). Furthermore, whereas the validation of survival prediction was largely consistent between the retrospective and prospective datasets, there was more variability with regard to ECMO and RRT. A possible reason for this might be structural differences between the retro- and prospective datasets, e.g. changes in treatment or age cohort over time. However, the moderate predictive capabilities of the variables used in these ML models leave open the opportunity to add further, even translational technologies for risk prediction in future. A strength of our approach is the ability to determine a weight for individual patient factors with respect to an individual prediction. Additionally, risk factors are presented with a shape function. This allows for a more detailed interpretation and segmentation of risk factors than a simple linear incrementation, as it is the case for the linear regression. Finally, due to the imbalanced dataset (more patients survived ICU therapy, more patients did not need ECMO or RRT), our model is more reliable for predicting “survival” than “mortality”. Nonetheless, the strength of these clinical data is the generalizability across institutions and even other similarly resourced countries.

## Conclusions

Yet, we present individual risk factors that can be combined for a prediction of “survival” during COVID-19 treatment and ICU course and these factors are weighed for importance. This has been done for the first time and will allow clinicians to weigh clinical criteria for outcome prediction in the patients treated.

## Supplementary Information

**Additional file 1**. Supplementary tables and figures.

## Data Availability

Data can be obtained from the authors upon reasonable request.

## Abbreviations

CI: Confidence interval
CV: Cross-validation
DIVI: German interdisciplinary association for intensive care and emergency medicine
EBM: Explainable boosting machine
ECMO: Extracorporeal membrane oxygenation
GAM: Generalized additive model
GCS: Glasgow Coma Scale
ICU: Intensive Care Unit
IQR: Inter-quartile range
PCR: Polymerase chain reaction
PEEP: Positive end expiratory pressure
RF: Random forest classifier
RRT: Renal replacement therapy
SD: Standard deviation
SOFA: Sequential organ failure assessment
SVC: Support vector classifier
