# Supplementary material for: Machine learning identifies ICU outcome predictors in a multicenter COVID-19 cohort
Source: Crit Care. 2021 Aug 17;25:295. doi: 10.1186/s13054-021-03720-4 (PMC8370055; doi:10.1186/s13054-021-03720-4)
Supplement: Supplementary file 1 — Additional file 1. Supplementary tables and figures. [file 13054_2021_3720_MOESM1_ESM.pdf]

Supplemental data

Machine Learning Identifies and Weighs Risk Factors Predicting Outcome in COVID-19 ICU Patients.

Supplemental Table E1: Table showing level of care at participating centers 3

Supplemental Table E2: Conversion table used to estimate FiO2 in patients breathing spontaneously with supplemental oxygen 6

Supplemental Table E3: Descriptive statistics of outlier data excluded 7

Supplemental Table E4: Clinical characteristics of retrospective (N=713) and prospective (N=475) patient cohort included in the study; therapeutic interventions, clinical and laboratory parameters 8

Supplemental Table E5: Clinical characteristics of patients who chose to discontinue ICU care; therapeutic interventions, clinical and laboratory parameters 14

Supplemental Table E6: Respiratory therapy and organ failure support in ICU patients......18

Supplemental Table E7: Description of the machine learning process..................................20

Variables excluded from the ML process due to more than 30% missing data 20

Supplemental Table E8: Variables used for the ML models 21

Supplemental Table E9: Overall performance of the machine learning models for ICU outcome prediction 23

Supplemental Figure E1: Map illustrating location of participating centers in Germany......24

Supplemental Figure E2: Patient selection chart...25

Supplemental Figure E3: Kaplan Meier Curves for probability of ICU survival 26

Supplemental Figure E4: PR-AUC curve for the EBM models (complete dataset) 27

Supplemental Figure E5: Comparison of shape functions of 5 selected variables of the EBM model for the prediction of “ICU survival” using the different datasets out of the 20 most important variables 29

References 33

Supplemental Table E1: Table showing level of care at participating centers

Hospital	Participating Department
**Tertiary Care University Hospitals**	
University Hospital RWTH Aachen, Aachen	Department of Intensive Care Medicine
Charité - Universitätsmedizin Berlin, corporate member of Freie Universität Berlin, Humboldt-Universität zu Berlin, and Berlin Institute of Health, Germany	Department of Anesthesiology and Operative Intensive Care Medicine (CCM, CVK)
Knappschaftskrankenhaus Bochum, Bochum	Department of Anesthesiology/Intensive Care Medicine/Pain Therapy
University Hospital Bonn, Bonn	Department of Anaesthesiology and Intensive Care Medicine
University Hospital Carl Gustav Carus, Technische Universität Dresden, Dresden	Department of Anesthesiology and Intensive Care Medicine
University Hospital Düsseldorf, Düsseldorf	Department of Anaesthesiology
University Hospital Essen, Essen	Department of Anesthesiology and Intensive Care Medicine
University Hospital Frankfurt, Goethe University, Frankfurt	Department of Anaesthesiology, Intensive Care Medicine and Pain Therapy
Medical Center - University of Freiburg, Freiburg	Department of Anesthesiology and Critical Care
University of Göttingen, Göttingen	Center for Anesthesiology, Emergency and Intensive Care Medicine

University Medicine Greifswald, Greifswald	Department of Anesthesiology
University Hospital Halle (Saale), Halle (Saale)	Cardiology, Angiology and Intensive Care Medicine
Heidelberg University Hospital, Heidelberg	Department of Anesthesiology
Leipzig University Hospital, Leipzig	Department of Anesthesiology and Intensive Care
University Medical Center Schleswig-Holstein, Campus Lübeck and University of Lübeck	Department of Anesthesiology and Intensive Care
Otto-von-Guericke-University Magdeburg, Magdeburg	Department of Anaesthesiology and Intensive Care Therapy
University Hospital Giessen and Marburg, and Philipps University Marburg, Marburg	Department of Anesthesiology and Intensive Care Medicine
University Hospital Giessen and Marburg, and Justus-Liebig University Giessen, Giessen	Department of Anesthesiology, Intensive Care Medicine and Pain Therapy
Klinikum rechts der Isar, Technical University of Munich	Department of Anesthesiology and Intensive Care
Saarland University Hospital Medical Center, Homburg/Saar	Department of Anesthesiology, Intensive Care Medicine and Pain Medicine
University Hospital Tübingen, Eberhard-Karls-University Tübingen, Tübingen	Department of Anaesthesiology and Intensive Care Medicine
Ulm University, Ulm	Department of Anesthesiology and Intensive Care Medicine

Cologne-Merheim Medical Centre, Witten/Herdecke University	Department of Anaesthesiology and Intensive Care Medicine
University Hospitals of Würzburg, University of Würzburg, Würzburg	Department of Anaesthesiology, Intensive Care, Emergency and Pain Medicine

**Primary and Secondary Care Hospitals**	
Zollernalb Klinikum, Balingen	Center for Anaesthesia, Intensive Care and Emergency Medicine
Evangelisches Krankenhaus Bethesda, Duisburg	Department of Anesthesiology and Intensive Care Medicine
St. Elisabethen Klinikum, Ravensburg.	Department for Anesthesiology, Intensive Care Medicine, Emergency Medicine and Pain Medicine

Enrolling centers

Retrospective study n=27

Prospective study n=10

Supplemental Table E2: Conversion table used to estimate FiO2 in patients breathing spontaneously with supplemental oxygen

**Oxygen Delivery Method and delivered O_2**	**Estimated F_iO_2**
Nasal cannula O_2	
2	0.28
3	0.32
4	0.36
5	0.40
6	0.44
Simple Face Mask O_2	
5	0.40
6-7	0.50
7-8	0.60
Non Re-Breather Face-Mask O_2	
6	0.60
7	0.70
8	0.80
9	0.90
10	0.95

Reference: The Extended Study of Prevalence of Infection in Intensive Care (EPIC II) study.

Supplemental Table E3: Descriptive statistics of outlier data excluded

**Parameter**	**Range accepted**	**Excluded data points (n)**	
		Retrospective cohort	*Prospective cohort*
Lymphocytes (n/nl)	0.1 to 50	26	51
Neutrophils (n/nl)	0.18 to 60	9	21
Leucocytes (n/nl)	1 to 100	3	3
Hemoglobin (g/dl)	5 to 20	0	6
Creatinine (mg/dl)	0 to 12	0	4
CRP (mg/dl)	0.01 to 80	1	0
Static compliance (ml/mbar)	0 to 100	11	6
PaO_2/FiO_2 Quotient	0 to 500	4	5
Driving Pressure (mbar)	0 to 30	3	0

Supplemental Table E4: Clinical characteristics of retrospective (N=713) and prospective (N=475) patient cohort included in the study; therapeutic interventions, clinical and laboratory parameters

	Retrospective cohort					Prospective cohort				
**Parameter**	**Total N**	**Missing N**	**non-survival**	**survival**	**total**	**Total N**	**Missing N**	**non-survival**	**survival**	**total**
Total N (%)			214 (30.0)	499 (70.0)	713			189 (39,8)	286 (60.2)	475
Age (years)	713	0	68.0 (58.0 to 76.0)	62.0 (53.0 to 72.0)	63.0 (54.0 to 74.0)	475	0	66.0 (58.0 to 73.0)	62.0 (53.0 to 71.0)	64.0 (54.0 to 72.5)
Age groups	713	0				475	0			
18 – 29 years			2 (0.9)	14 (2.8)	16 (2.2)			0 (0.0)	12 (4.2)	12 (2.5)
30 – 39 years			3 (1.4)	26 (5.2)	29 (4.1)			5 (2.6)	11 (3.8)	16 (3.4)
40 – 49 years			14 (6.5)	50 (10.0)	64 (9.0)			18 (9.5)	25 (8.7)	43 (9.1)
50 – 59 years			42 (19.6)	125 (25.1)	167 (23.4)			33 (17.5)	71 (24.8)	104 (21.9)
60 – 69 years			62 (29.0)	127 (25.5)	189 (26.5)			68 (36.0)	75 (26.2)	143 (30.1)
70 – 79 years			56 (26.2)	114 (22.8)	170 (23.8)			49 (25.9)	75 (26.2)	124 (26.1)
80 – 89 years			34 (15.9)	40 (8.0)	74 (10.4)			14 (7.4)	17 (5.9)	31 (6.5)
> 90 years			1 (0.5)	3 (0.6)	4 (0.6)			2 (1.1)	0 (0.0)	2 (0.4)
Sex	713	0				475	0			
female			39 (18.2)	149 (29.9)	188 (26.4)			53 (28.0)	92 (32.2)	145 (30.5)
male			175 (81.8)	350 (70.1)	525 (73.6)			136 (72.0)	194 (67.8)	330 (69.5)
BMI (kg/m²)	660	53	28.0 (24.9 to 33.9)	28.3 (25.4 to 32.7)	28.2 (25.2 to 33.1)	462	13	28.4 (25.4 to 32.7)	28.4 (24.9 to 32.7)	28.4 (25.1 to 32.7)
BMI groups	660	53				462	13			

below 20 kg/m²			2 (1.0)	9 (2.0)	11 (1.7)			6 (3.3)	7 (2.5)	13 (2.8)
20 – 25 kg/m²			50 (24.6)	102 (22.3)	152 (23.0)			38 (20.8)	63 (22.6)	101 (21.9)
25 – 30 kg/m²			75 (36.9)	170 (37.2)	245 (37.1)			66 (36.1)	97 (34.8)	163 (35.3)
above 30 kg/m²			76 (37.4)	176 (38.5)	252 (38.2)			73 (39.9)	112 (40.1)	185 (40.0)
Bloodgroup	435	278				321	154			
0			62 (40.0)	94 (33.6)	156 (35.9)			59 (40.4)	64 (36.6)	123 (38.3)
A			63 (40.6)	141 (50.4)	204 (46.9)			61 (41.8)	72 (41.1)	133 (41.4)
AB			4 (2.6)	13 (4.6)	17 (3.9)			6 (4.1)	12 (6.9)	18 (5.6)
B			26 (16.8)	32 (11.4)	58 (13.3)			20 (13.7)	27 (15.4)	47 (14.6)
**Past medical history and chronic medications**										
Arterial hypertension	713	0	144 (67.3)	314 (62.9)	458 (64.2)	475	0	111 (58.7)	167 (58.4)	278 (58.5)
Cardiovascular disease	713	0	79 (36.9)	120 (24.0)	199 (27.9)	475	0	45 (23.8)	68 (23.8)	113 (23.8)
Chronic arrhythmia	713	0	37 (17.3)	51 (10.2)	88 (12.3)	475	0	24 (12.7)	34 (11.9)	58 (12.2)
COPD	713	0	19 (8.9)	42 (8.4)	61 (8.6)	475	0	19 (10.1)	27 (9.4)	46 (9.7)
Other lung disease	713	0	23 (10.7)	52 (10.4)	75 (10.5)	475	0	21 (11.1)	28 (9.8)	49 (10.3)
Nicotine abuse	713	0	22 (10.3)	43 (8.6)	65 (9.1)	475	0	14 (7.4)	35 (12.2)	49 (10.3)
History of solid organ transplant	713	0	3 (1.4)	5 (1.0)	8 (1.1)	475	0	6 (3.2)	9 (3.1)	15 (3.2)
History of bone marrow transplant	713	0	2 (0.9)	2 (0.4)	4 (0.6)	475	0	1 (0.5)	3 (1.0)	4 (0.8)
Alcoholism	713	0	5 (2.3)	11 (2.2)	16 (2.2)	475	0	8 (4.2)	12 (4.2)	20 (4.2)

Chronic kidney failure	713	0	33 (15.4)	66 (13.2)	99 (13.9)	475	0	20 (10.6)	27 (9.4)	47 (9.9)
Diabetes mellitus	713	0	58 (27.1)	131 (26.3)	189 (26.5)	475	0	68 (36.0)	88 (30.8)	156 (32.8)
NIDDM	713	0	42 (19.6)	83 (16.6)	125 (17.5)	475	0	46 (24.3)	50 (17.5)	96 (20.2)
Prior thrombotic events[1]	713	0	9 (4.2)	23 (4.6)	32 (4.5)	475	0	15 (7.9)	12 (4.2)	27 (5.7)
ACE inhibitors	713	0	46 (21.5)	84 (16.8)	130 (18.2)	475	0	49 (25.9)	89 (31.1)	138 (29.1)
AT2 receptor blocker	713	0	25 (11.7)	81 (16.2)	106 (14.9)	475	0	22 (11.6)	37 (12.9)	59 (12.4)
Beta blockers	713	0	66 (30.8)	140 (28.1)	206 (28.9)	475	0	50 (26.5)	88 (30.8)	138 (29.1)
Anti-platelet medication	713	0	56 (26.2)	100 (20.0)	156 (21.9)	475	0	39 (20.6)	65 (22.7)	104 (21.9)
NOAC	713	0	11 (5.1)	31 (6.2)	42 (5.9)	475	0	18 (9.5)	26 (9.1)	44 (9.3)
Corticosteroids	713	0	26 (12.1)	31 (6.2)	57 (8.0)	475	0	18 (9.5)	32 (11.2)	50 (10.5)
Immunosuppressive drugs	713	0	9 (4.2)	15 (3.0)	24 (3.4)	475	0	12 (6.3)	16 (5.6)	28 (5.9)
Opioids	713	0	8 (3.7)	21 (4.2)	29 (4.1)	475	0	11 (5.8)	17 (5.9)	28 (5.9)
**Status at ICU admission**										
Admission/Transfer status	713	0				475	0			
External transfer			111 (51.9)	249 (49.9)	360 (50.5)			95 (50.3)	87 (30.4)	182 (38.3)
Internal or direct admission			103 (48.1)	250 (50.1)	353 (49.5)			94 (49.7)	199 (69.6)	293 (61.7)
Ventilatory status at admission	713	0				475	0			
intubated			122 (57.0)	202 (40.5)	324 (45.4)			95 (50.3)	76 (26.6)	171 (36.0)

non-invasive assisted ventilation			19 (8.9)	55 (11.0)	74 (10.4)			21 (11.1)	37 (12.9)	58 (12.2)
spontaneous breathing			73 (34.1)	242 (48.5)	315 (44.2)			73 (38.6)	173 (60.5)	246 (51.8)
Prior days of non-invasive ventilation	575	138	0.0 (0.0 to 0.0)	0.0 (0.0 to 0.0)	0.0 (0.0 to 0.0)	442	33	0.0 (0.0 to 2.0)	0.0 (0.0 to 0.0)	0.0 (0.0 to 1.0)
Days prior invasive ventilation	644	69	0.0 (0.0 to 4.0)	0.0 (0.0 to 1.0)	0.0 (0.0 to 2.0)	457	18	0.0 (0.0 to 1.0)	0.0 (0.0 to 0.0)	0.0 (0.0 to 1.0)
RASS	654	59	-3 (-5 to 0)	0 (-4 to 0)	-1 (-4 to 0)	448	27	-1 (-5 to 0)	0 (-2 to 0)	0 (-5 to 0)
SOFA (w/o GCS)	713	0	7 (5 to 10)	5 (3 to 7)	6 (4 to 8)	475	0	6 (4 to 8)	4 (3 to 6)	4 (3 to 7)
Murray Lung Injury Score	697	16	3.0 (2.5 to 3.5)	3.0 (2.3 to 3.5)	3.0 (2.5 to 3.5)	461	14	3.5 (3.0 to 3.6)	3.0 (2.1 to 3.5)	3.0 (2.5 to 3.5)
ARDS grading according to PaO2/FiO2 quotient	705	8				454	21			
Mild (PaO2/FiO2 201 to 300)			21 (9.9)	96 (19.6)	117 (16.7)			24 (13.0)	52 (19.3)	76 (16.7)
Moderate (PaO2/FiO2 101 to 200)			108 (50.9)	234 (47.8)	342 (48.7)			81 (43.8)	124 (46.1)	205 (45.2)
Severe (PaO2/FiO2 <= 100)			73 (34.4)	116 (23.7)	189 (26.9)			69 (37.3)	70 (26.0)	139 (30.6)
no ARDS			10 (4.7)	44 (9.0)	54 (7.7)			11 (5.9)	23 (8.6)	34 (7.5)
Static compliance (ml/mbar)[2]	411	302	36.5 (27.5 to 47.7)	38.7 (30.0 to 50.0)	37.8 (29.0 to 49.9)	201	274	31.7 (23.0 to 40.0)	35.2 (28.1 to 46.0)	33.3 (24.4 to 41.8)
Driving pressure (mbar)[2]	422	291	13.0 (10.0 to 16.0)	12.0 (10.0 to 15.0)	13.0 (10.0 to 15.0)	214	261	13.0 (10.0 to 16.0)	12.0 (10.0 to 15.0)	12.0 (10.0 to 15.0)
Hemoglobin (g/dl)	709	4	11.4 (9.7 to 13.0)	11.7 (9.9 to 13.1)	11.6 (9.8 to 13.1)	472	3	10.6 (9.4 to 12.6)	12.4 (10.5 to 13.7)	11.6 (10.0 to 13.3)

Platelets (x10³µl-1)	710	3	199.0 (146.0 to 273.0)	230.0 (177.0 to 313.0)	223.0 (166.0 to 299.0)	470	5	207.5 (147.0 to 287.2)	239.0 (177.0 to 321.0)	226.5 (163.0 to 310.2)
Leucocytes (n/nl)	710	3	10.2 (6.7 to 13.5)	8.7 (5.9 to 11.8)	9.0 (6.2 to 12.5)	472	3	11.1 (8.0 to 16.2)	9.2 (6.4 to 12.4)	10.0 (6.8 to 14.1)
Lymphocytes (n/nl)	612	101	0.8 (0.4 to 1.1)	0.8 (0.6 to 1.2)	0.8 (0.6 to 1.2)	365	110	0.7 (0.4 to 1.2)	0.8 (0.5 to 1.2)	0.8 (0.5 to 1.2)
Neutrophiles (n/nl)	544	169	8.2 (5.0 to 11.5)	6.5 (4.4 to 9.1)	7.0 (4.7 to 9.9)	349	126	9.4 (6.4 to 14.2)	7.3 (4.7 to 10.0)	8.0 (5.3 to 11.5)
Platelet/neutrophile ratio	542	171	24.4 (17.4 to 37.3)	34.4 (25.2 to 55.3)	32.4 (22.7 to 50.7)	347	128	21.5 (12.2 to 34.2)	34.3 (23.1 to 49.9)	28.7 (18.0 to 43.7)
Platelet/lymphocyte ratio	609	104	285.5 (168.4 to 436.4)	279.4 (184.2 to 425.9)	280.8 (178.9 to 432.3)	362	113	256.4 (139.4 to 503.8)	285.7 (178.8 to 434.8)	280.5 (168.3 to 470.7)
C-reactive protein (mg/dl)	697	16	18.2 (10.4 to 30.4)	15.0 (8.9 to 23.6)	16.0 (9.4 to 26.6)	445	30	15.6 (8.5 to 23.9)	12.2 (5.8 to 20.1)	13.3 (6.6 to 22.0)
Procalcitonin (ng/ml)	688	25	1.0 (0.3 to 2.9)	0.4 (0.1 to 1.1)	0.5 (0.2 to 1.6)	467	8	0.6 (0.2 to 1.7)	0.2 (0.1 to 0.7)	0.3 (0.1 to 1.1)
Interleukin-6 (pg/ml)	527	186	206.0 (84.6 to 571.2)	98.4 (45.0 to 226.5)	120.0 (54.1 to 335.0)	325	150	111.0 (50.4 to 295.8)	61.4 (25.0 to 127.0)	82.2 (32.9 to 187.0)
Ferritin (µg/dl)	449	264	161.8 (94.4 to 304.5)	121.3 (57.1 to 201.0)	129.4 (68.7 to 216.6)	174	301	138.5 (82.3 to 249.4)	100.6 (60.6 to 157.8)	115.0 (69.5 to 184.9)
D-Dimer (µg/ml)	558	155	4.0 (1.5 to 11.0)	2.5 (1.2 to 5.1)	2.8 (1.3 to 6.6)	348	127	5.4 (1.9 to 18.9)	1.7 (0.9 to 4.7)	2.9 (1.2 to 10.6)
Total bilirubin (mg/dl)	700	13	0.7 (0.4 to 1.2)	0.6 (0.4 to 0.9)	0.6 (0.4 to 1.0)	462	13	0.7 (0.4 to 1.1)	0.5 (0.3 to 0.7)	0.6 (0.4 to 0.8)

Creatinine (mg/dl)	713	0	1.4 (1.0 to 2.5)	1.0 (0.7 to 1.4)	1.1 (0.8 to 1.7)	467	8	1.1 (0.7 to 1.8)	0.9 (0.8 to 1.3)	1.0 (0.8 to 1.5)
**ICU outcomes**										
Mortality n/%			214 (30.0)					186 (39.4)		
LOS ICU (days)	713	0	13.0 (6.2 to 25.0)	18.0 (8.0 to 37.0)	17.0 (7.0 to 33.0)	475	0	15.0 (10.0 to 23.0)	12.0 (5.0 to 27.0)	14.0 (7.0 to 25.5)
Transfer destination	518	195				472	3			
intermediate care			na	36 (7.3)	na			na	15 (5.3)	15 (3.2)
normal ward			na	282 (57.1)	na			3 (1.6)	207 (73.1)	210 (44.5)
other ICU			na	95 (19.2)	na			na	30 (10.6)	30 (6.4)
REHA			na	81 (16.4)	na			na	31 (11.0)	31 (6.6)

All values are reported as absolute numbers and percentages for categorical variables, and as median and interquartile ranges (IQR) if not distributed normally for continuous variables.

Abbreviations: BMI: body-mass-index (kg/m²); COPD: chronic-obstructive pulmonary disease; ICU: intensive care unit; LOS: length-of-stay; NIDDM: Non-insulin dependent diabetes mellitus; NOAC: novel oral anticoagulants; RASS: Richmond agitation sedation scale; SOFA score without GCS: sequential organ failure assessment score without Glasgow coma scale (GCS).

[1] Prior thrombotic events: e.g. deep vein thrombosis, pulmonary embolism.

[2] At ICU admission or first value after intubation at day of ICU admission.

Supplemental Table E5: Clinical characteristics of patients who chose to discontinue ICU care; therapeutic interventions, clinical and laboratory parameters

Parameter	Total N	Missing N		All patients
Total N (%)				107 (100.0)
Age	107	0	Mean (SD)	73.8 (10.1)
Age groups	107	0		
18 – 29				0 (0.0)
30 – 39 years				0 (0.0)
40 – 49 years				0 (0.0)
50 – 59 years				11 (10.3)
60 – 69 years				24 (22.4)
70 – 79 years				35 (32.7)
80 – 89 years				33 (30.8)
90 – 99 years				4 (3.7)
Sex	107	0		
female				32 (29.9)
male				75 (70.1)
BMI	100	7	Mean (SD)	27.2 (5.8)
BMI groups	100	7		
below 20 kg/m²				6 (6.0)
20 – 25 kg/m²				37 (37.0)
25 – 30 kg/m²				34 (34.0)
above 30 kg/m²				23 (23.0)

Blood group	79	28	
0			29 (36.7)
A			38 (48.1)
AB			5 (6.3)
B			7 (8.9)
Arterial hypertension	107	0	78 (72.9)
Cardiovascular disease	107	0	34 (31.8)
Chronic arrhythmia	107	0	18 (16.8)
COPD	107	0	8 (7.5)
Other lung diseases	107	0	12 (11.2)
Nicotine abuse	107	0	6 (5.6)
History of solid organ transplant	107	0	2 (1.9)
History of bone marrow transplant	107	0	1 (0.9)
Alcoholism	107	0	3 (2.8)
Chronic kidney failure	107	0	20 (18.7)
Diabetes mellitus	107	0	29 (27.1)
NIDDM	107	0	18 (16.8)
Prior thrombotic events	107	0	10 (9.3)
ACE inhibitors	107	0	32 (29.9)
AT2 receptor blocker	107	0	15 (14.0)
Beta blockers	107	0	43 (40.2)
Anti-platelet medication	107	0	39 (36.4)

Novel oral anticoagulants (NOAC)	107	0		11 (10.3)
Corticoids	107	0		8 (7.5)
Immunosuppressive drugs	107	0		6 (5.6)
Opioids	107	0		7 (6.5)
Admission/Transfer status	107	0		
External Transfer				37 (34.6)
Internal Admission				70 (65.4)
Ventilatory status at admission	107	0		
Intubated				39 (36.4)
Non-invasive assisted ventilation				15 (14.0)
spontaneous breathing				53 (49.5)
Prior days of non-invasive ventilation	90	17	Median (IQR)	0.0 (0.0 to 0.0)
Prior days of invasive ventilation	96	11	Median (IQR)	0.0 (0.0 to 0.0)
RASS	105	2	Median (IQR)	0 (-5 to 0)
SOFA (w/o GCS)	107	0	Median (IQR)	6.0 (4.0 to 8.0)
Murray Lung Injury Score	106	1	Median (IQR)	3.1 (2.3 to 3.5)
ARDS grading according to PaO2/FiO2 Quotient	59	0		
no ARDS				13 (12.1)
PaO2/FiO2 > 300				
Mild				17 (15.9)
PaO2/FiO2 201-300				
Moderate				45 (42.1)
PaO2/FiO2 101-200				
Severe				32 (29.9)
PaO2/FiO2 < 100				

Static compliance (ml/mbar)	56	51	Mean (SD)	33.4 (15.6)
Driving pressure (mbar)	56	51	Mean (SD)	13.4 (4.2)
Hemoglobin (g/dl)	105	2	Mean (SD)	11.2 (2.4)
Platelets (x10³µl-1)	105	2	Median (IQR)	199.0 (138.0 to 277.0)
Leucocytes (n/nl)	104	3	Median (IQR)	9.7 (6.8 to 13.6)
Lymphocytes (n/nl)	83	24	Median (IQR)	0.6 (0.5 to 1.0)
Neutrophiles (n/nl)	82	25	Mean (SD)	8.6 (4.5)
Platelet/neutrophile ratio	82	25	Median (IQR)	27.1 (18.2 to 37.3)
Platelet/lymphocyte ratio	82	25	Median (IQR)	305.4 (175.3 to 491.4)
C-reactive protein (mg/dl)	100	7	Median (IQR)	13.4 (7.3 to 23.4)
Procalcitonin (ng/ml)	102	5	Median (IQR)	0.4 (0.1 to 1.9)
Interleukin-6 (pg/ml)	68	39	Median (IQR)	119.0 (39.6 to 408.8)
Ferritin (µg/dl)	58	49	Median (IQR)	108.3 (59.8 to 210.7)
D-Dimer (µg/ml)	84	23	Median (IQR)	4.1 (1.5 to 8.6)
Total bilirubin (mg/dl)	104	3	Median (IQR)	0.6 (0.4 to 0.9)
Creatinine (mg/dl)	105	2	Median (IQR)	1.2 (0.8 to 1.9)
LOS ICU (days)				15.0 (7.0 to 24.0)
ICU discharge status	107	0	non-survival	95 (88.8)

ARDS: acquired respiratory distress syndrome, grading according to Berlin definition; BMI: body-mass-index (kg/m²); COPD: chronic-obstructive pulmonary disease; ICU: intensive care unit; LOS: length-of-stay; NIDDM: Non-insulin dependent diabetes mellitus; NOAC: novel oral anticoagulants; RASS: Richmond agitation sedation scale; SOFA score without GCS: sequential organ failure assessment score without Glasgow coma scale (GCS).

[1] Prior thrombotic events: e.g. deep vein thrombosis, pulmonary embolism.

Supplemental Table E6: Respiratory therapy and organ failure support in ICU patients

	Retrospective cohort				Prospective cohort			
**Parameter**	**Total N**	**Missing N**	**non-survival**	**survival**	**Total N**	**Missing N**	**non-survival**	**survival**
Total N (%)			214 (30.0)	499 (70.0)			189 (40.0)	284 (60.0)
Intubation and mechanical ventilation during ICU stay	713	0	194 (90.7)	369 (73.9)	473	0	184 (97.4)	156 (54.9)
Reason for intubation	713	0			473	0		
Hypoxemia			176 (82.2)	331 (66.3)			158 (83.6)	133 (46.8)
Hypercapnia			44 (20.6)	81 (16.2)			37 (19.6)	23 (8.1)
Dyspnea			81 (37.9)	143 (28.7)			33 (17.5)	31 (10.9)
Fatigue			60 (28.0)	103 (20.6)			59 (31.2)	36 (12.7)
Public health indication (e.g. prevention of aerosol spread)			31 (14.5)	39 (7.8)			n/a	1 (0.4)
Prone positioning	713	0	154 (72.0)	270 (54.1)	473	0	158 (83.6)	125 (44.0)
Tracheostomy	713	0	81 (37.9)	198 (39.7)	473	0	58 (30.7)	80 (28.2)
Duration of invasive ventilation (days)	564	149	15.0 (22.0)	18.0 (24.5)	340	133	16.0 (14.0)	18.0 (29.0)
Duration of non-invasive ventilation (days)	213	500	1.5 (2.0)	4.0 (5.0)	200	273	3.0 (4.0)	5.0 (7.0)
ECMO therapy	713	0	83 (38.8)	67 (13.4)	473	0	86 (45.5)	25 (8.8)
duration (days)			11.5 (5.8 to 23.5)	16 (10 to 29)			18 (9.5 to 25.5)	17 (11.3 to 25.3)
CRRT or dialysis	713	0	149 (69.6)	160 (32.1)	473	0	125 (66.1)	48 (16.9)
Convalescent plasma therapy	627	86	7 (3.6)	5 (1.1)	448	25	9 (5.0)	7 (2.6)
Pulmonary embolism	713	0	30 (14.0)	27 (5.4)	473	0	22 (11.6)	24 (8.5)

Bacterial superinfection with pneumonia	713	0	127 (59.3)	217 (43.5)	473	0	100 (52.9)	85 (29.9)
Antibiotic therapy	713	0	204 (95.3)	410 (82.2)	473	0	167 (88.4)	187 (65.8)
Therapeutic dose unfractionated heparin	713	0	143 (66.8)	256 (51.3)	473	0	151 (79.9)	135 (47.5)
Therapeutic dose argatroban	713	0	30 (14.0)	62 (12.4)	473	0	27 (14.3)	35 (12.3)
Intravenous corticosteroids	713	0	108 (50.5)	107 (21.4)	473	0	169 (89.4)	220 (77.5)

All values are reported as median and interquartile ranges.

Abbreviations: BMI: body-mass-index (kg/m²); CRRT: continuous renal replacement therapy; ECMO: extra-corporeal membrane oxygenation; ICU: intensive care unit; LOS: length-of-stay; n/a not available.

Supplemental Table E7: Description of the machine learning process

Software used for the machine learning process and for visualization.<br><br>ML was performed in python (version 3.8.5) with jupyter notebook (jupyter-core 4.63 and jupyter notebook 6.1.5) and pandas (version 1.1.4) [1]. Standard implementations from scikit-learn (version 0.23.2) were used for Random Forest, Support-Vector-Classifier (SVC), and Cross-Validation (CV) [2]. For the EBM, we used Microsoft's open source implementation interpretML (Microsoft Corporation, Redmond, USA, version 0.2.4) [3]. For the visualization of the PR-AUC curves we used matplotlib (version 3.3.3). For all other visualizations, we used R (version 4.0.3) within RStudio (version 1.3.1093); tidyverse (version 1.3.0), latex2exp (version 0.4.0), ggpubr (version 0.4.0), patchwork (version 1.1.0), for the generation of tables finalfit (version 1.0.2), for Kaplan-Meier-Curves finalfit, survminer (version 0.4.8), and survival (version 3.2.7).
Variable representation<br><br>We transformed the following laboratory values to their log10(x+1)-scale (based on visual inspection) prior to their use as ML variables: lymphocytes, leucocytes, neutrophils, creatinine, procalcitonin, d-dimers, IL-6, ferritin, platelets, platelets/neutrophils ratio, platelets/lymphocytes ratio, bilirubin.
Variables excluded from the ML process due to more than 30% missing data<br><br>PEEP at admission<br><br>Compliance and Driving pressure at admission<br><br>Blood group<br><br>Ferritin at admission

Supplemental Table E8: Variables used for the ML models

**ML “admission” model**
Age
Sex
Admission/Transfer status
Admission from
Height
Weight
BMI
Arterial Hypertension
Cardiovascular Disease
Chronic arrhythmia
COPD
Other lung disease
Nicotine
H/O Solid Organ Transplant
H/O Bone Marrow Transplant
Alcoholism
Chronic kidney failure
Chronic dialysis
Diabetes mellitus
NIDDM
Prior thrombotic events
ACE inhibitors
AT2 receptor blocker
Beta blockers
Anti-platelet medication
NOAC
Corticoids
Immunosuppressive drugs
Opioids
Breathing status
Alveolar consolidation (quadrants,CXR)
Hypotension according to SOFA grading
Platelets
Total bilirubin
Creatinine
Murray lung injury score
SOFA score w/o GCS
Procalcitonin
Interleukin-6
Hemoglobin
D-Dimer
Prior days of invasive ventilation
Platelet/lymphocyte ratio
Platelet/neutrophile ratio
PaO2/FiO2 quotient at admission

Leucocytes
Lymphocytes (absolute)
Neutrophiles (absolute)
C-reactive protein

Supplemental Table E9: Overall performance of the machine learning models for ICU outcome prediction

a) Retrospective cohort

**Prediction variable**	**ICU survival** N=596		**ECMO therapy** N=596		**Renal Replacement Therapy** N=572	
**ML model**	Balanced accuracy	PR-AUC	Balanced accuracy	PR-AUC	Balanced accuracy	PR-AUC
RF	0.6	0.85	0.66	0.47	0.71	0.72
SVC	0.64	0.83	0.71	0.48	0.7	0.71
EBM	0.57		0.61		0.7	
EBM (10 interactions)	0.61	0.83	0.66	0.48	0.69	0.8

b) Prospective cohort

**Prediction variable**	**ICU survival** N=443		**ECMO therapy** N=443		**Renal Replacement Therapy** N=428	
**ML model**	Balanced accuracy	PR-AUC	Balanced accuracy	PR-AUC	Balanced accuracy	PR-AUC
RF	0.6	0.88	0.6	0.93	0.69	0.48
SVC	0.67	0.78	0.79	0.83	0.66	0.52
EBM	0.58		0.55		0.69	
EBM (10 interactions)	0.58	0.89	0.61	0.93	0.69	0.54

Supplemental Figure E1: Map illustrating location of participating centers in Germany

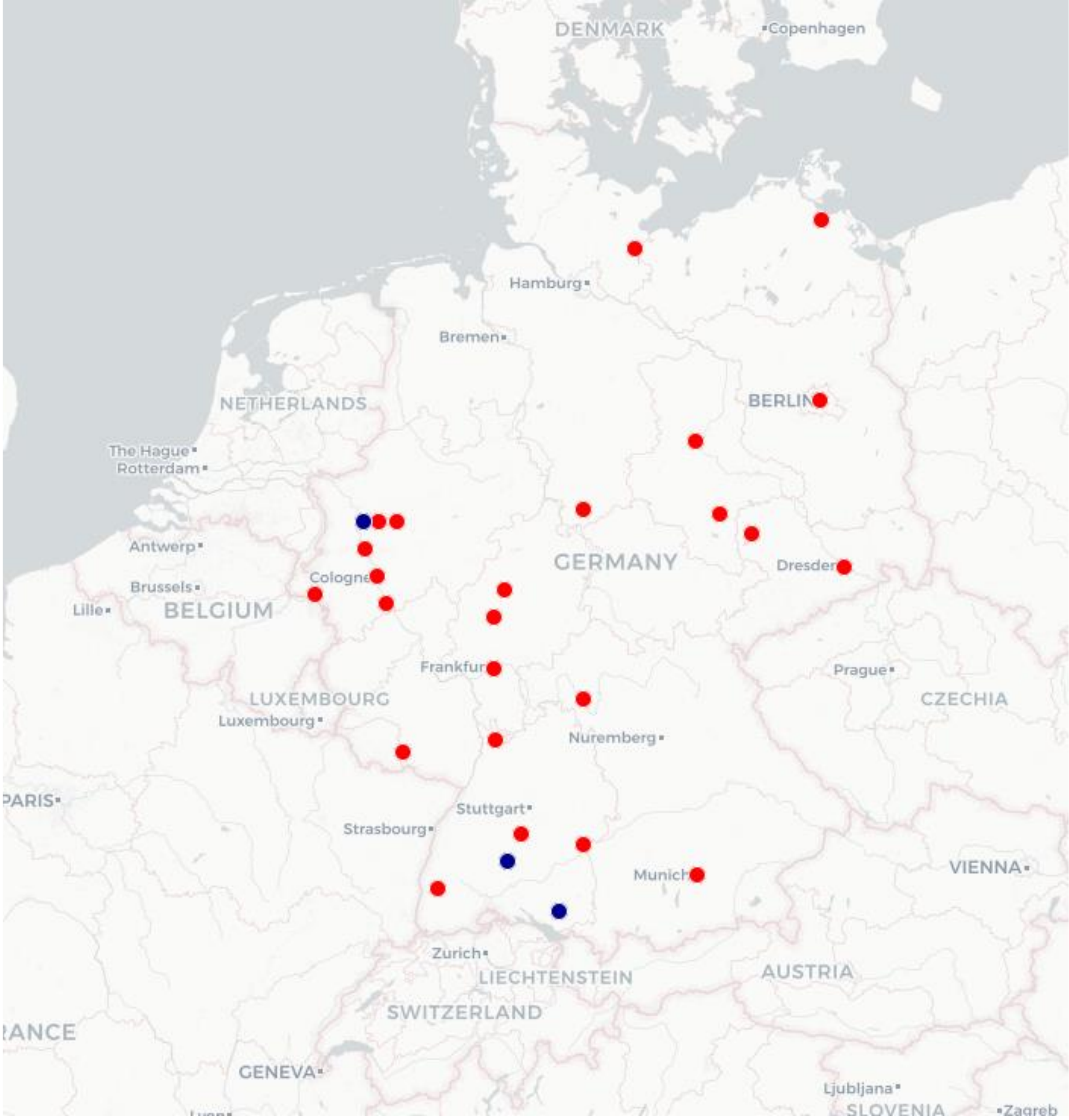

Map tiles by CartoDB, under CC BY 3.0. map data © OpenStreetMap contributors under ODbL

Red dots: Participating tertiary care university hospitals

Blue dots: Participating primary and secondary care hospitals

Supplemental Figure E2: Patient selection chart

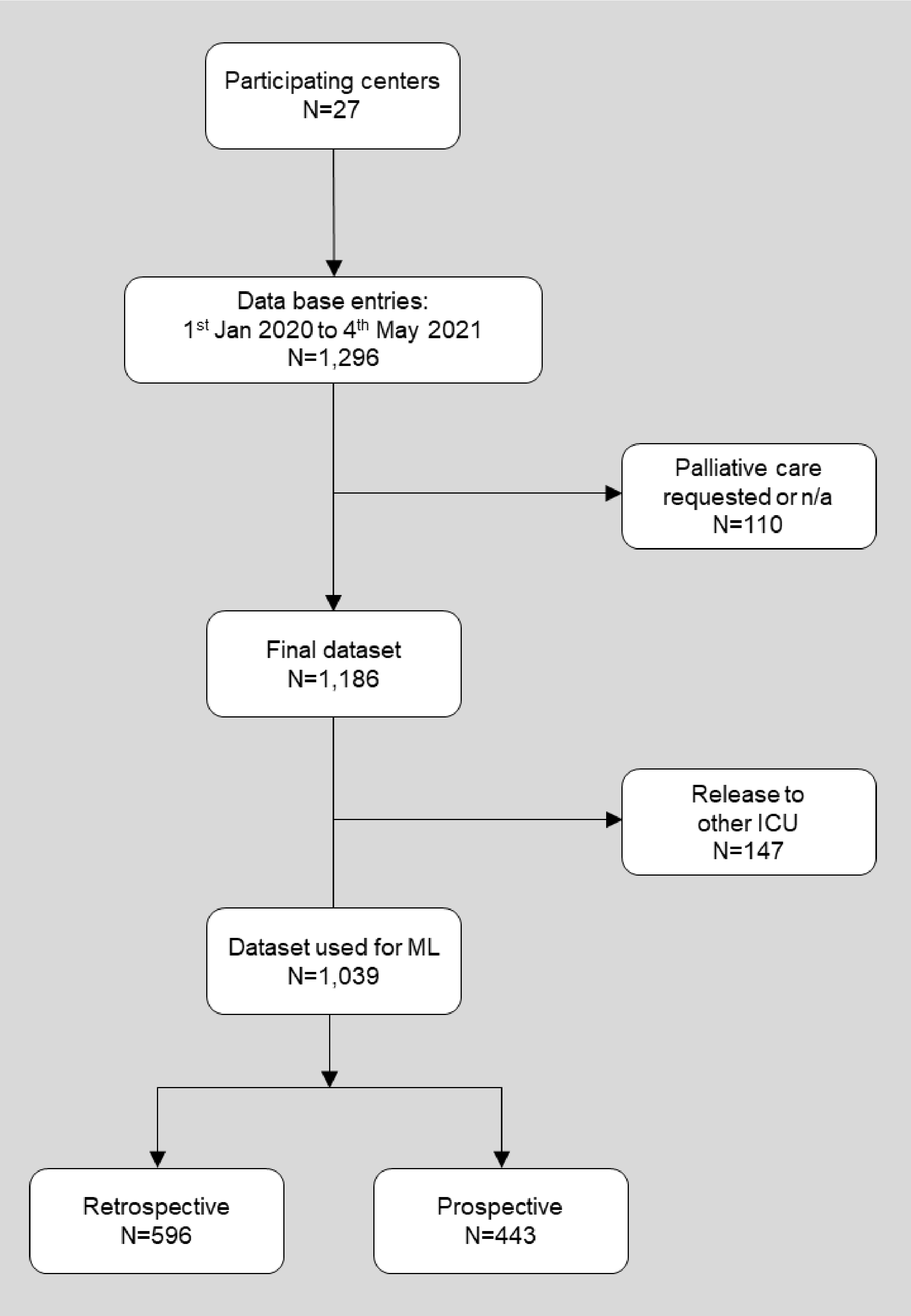

Abbreviations: ML machine learning; n/a not available.

Supplemental Figure E3: Kaplan Meier Curves for probability of ICU survival

a) ICU survival according to age groups

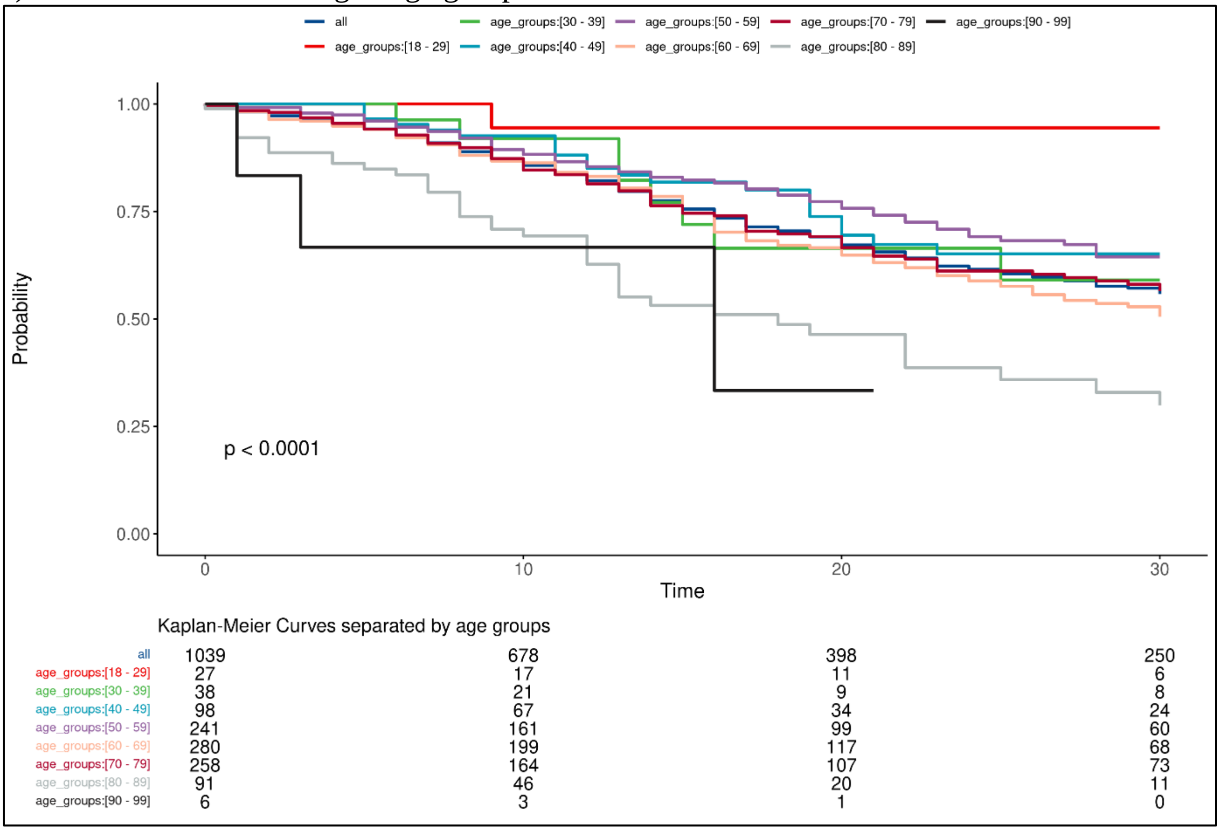

b) ICU survival according to ARDS severity

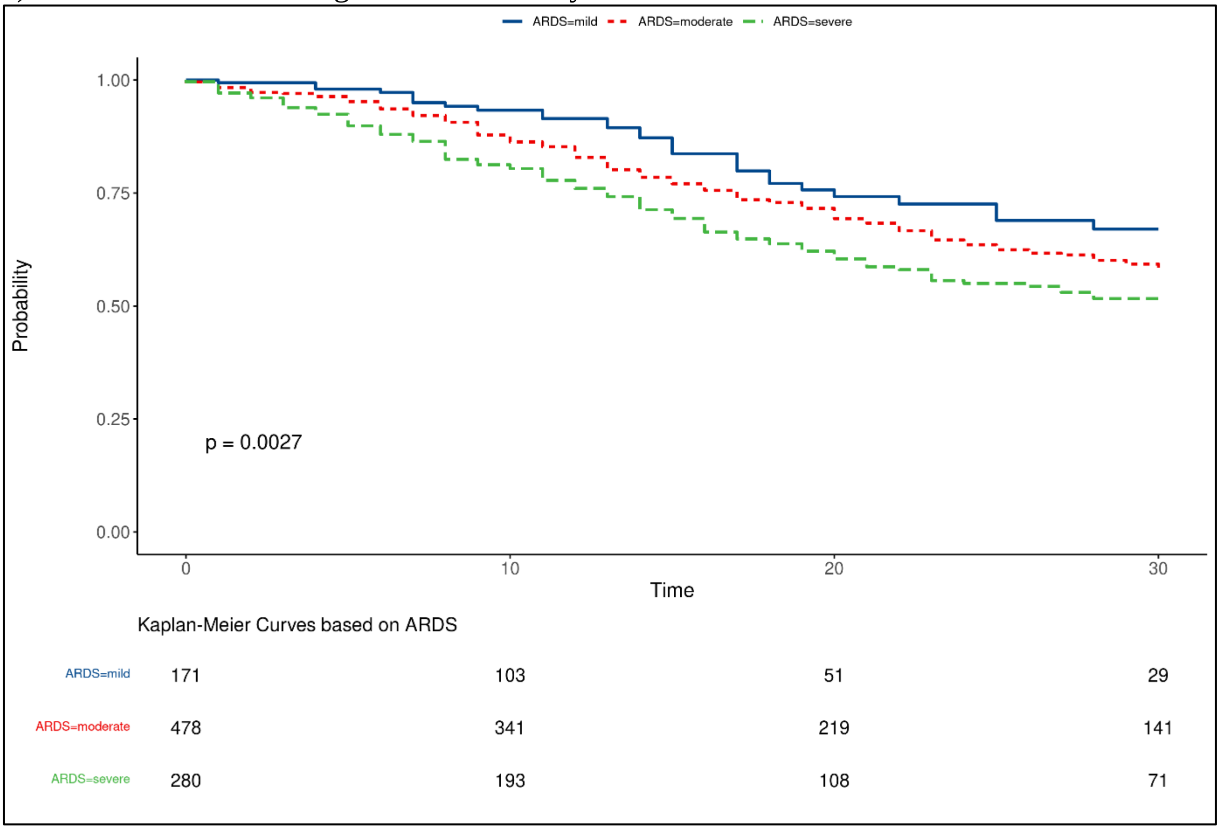

Supplemental Figure E4: PR-AUC curve for the EBM models (complete dataset)

a) EBM model (10-interactions) for prediction of ICU survival

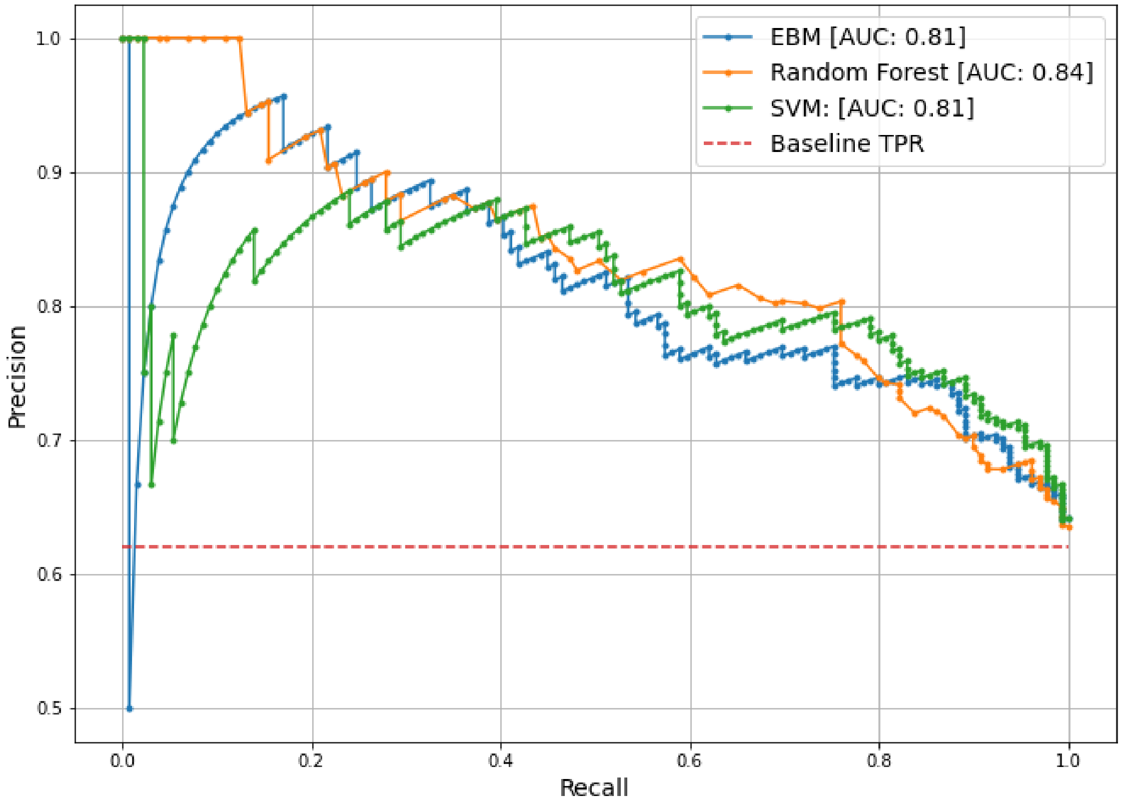

b) EBM model (10-interactions) for prediction of ECMO therapy

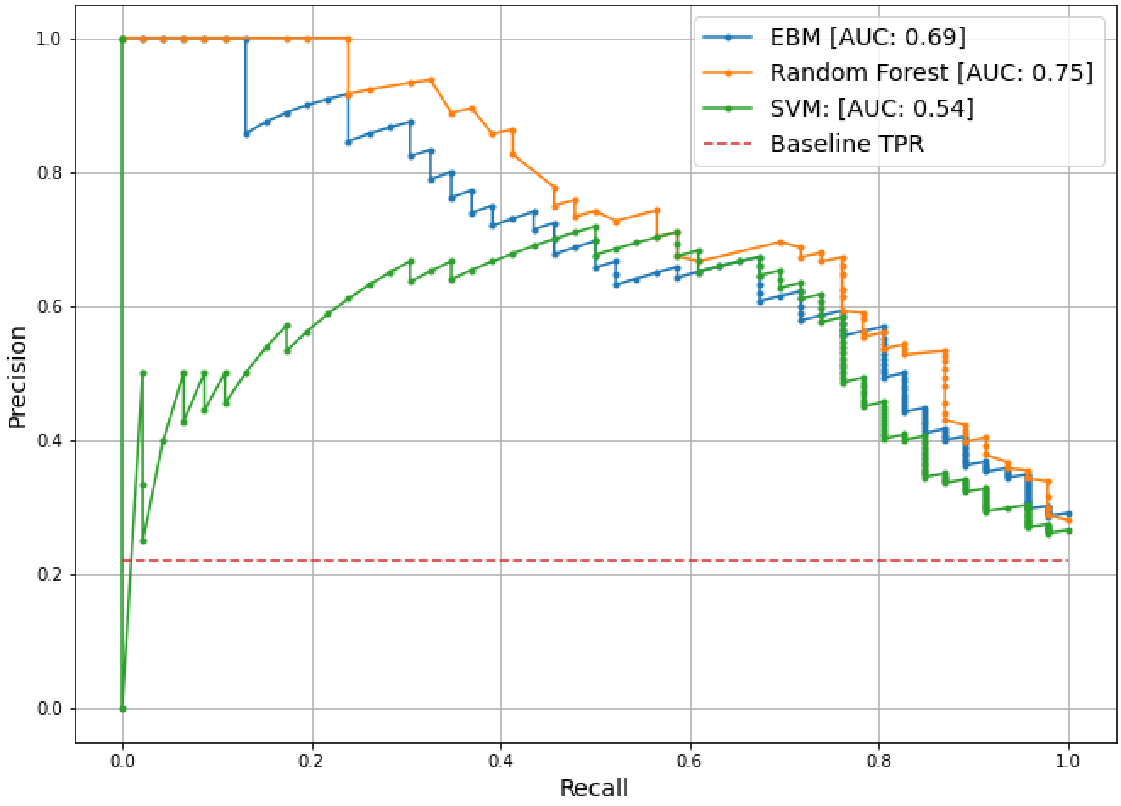

c) EBM model (10-interactions) for prediction of renal replacement therapy during ICU stay

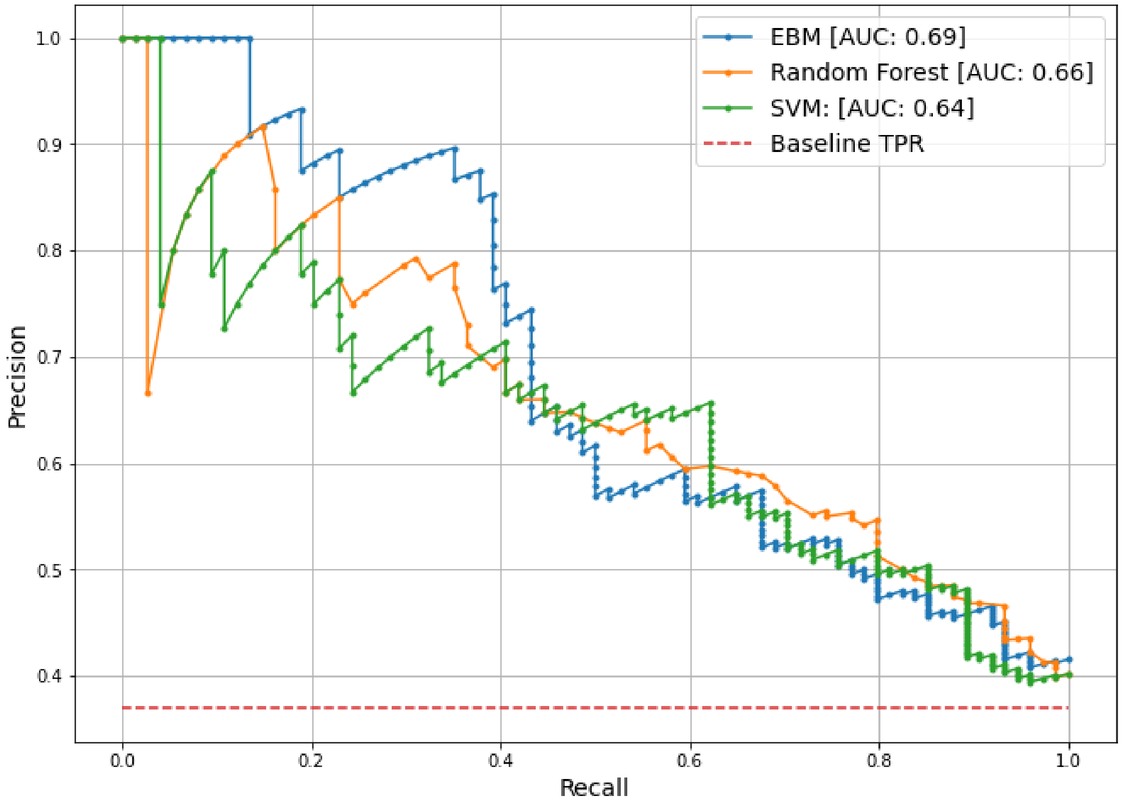

Supplemental Figure E5: Comparison of shape functions of 5 selected variables of the EBM model for the prediction of “ICU survival” using the different datasets out of the 20 most important variables

a) EBM model (10-interactions) for prediction of ICU survival

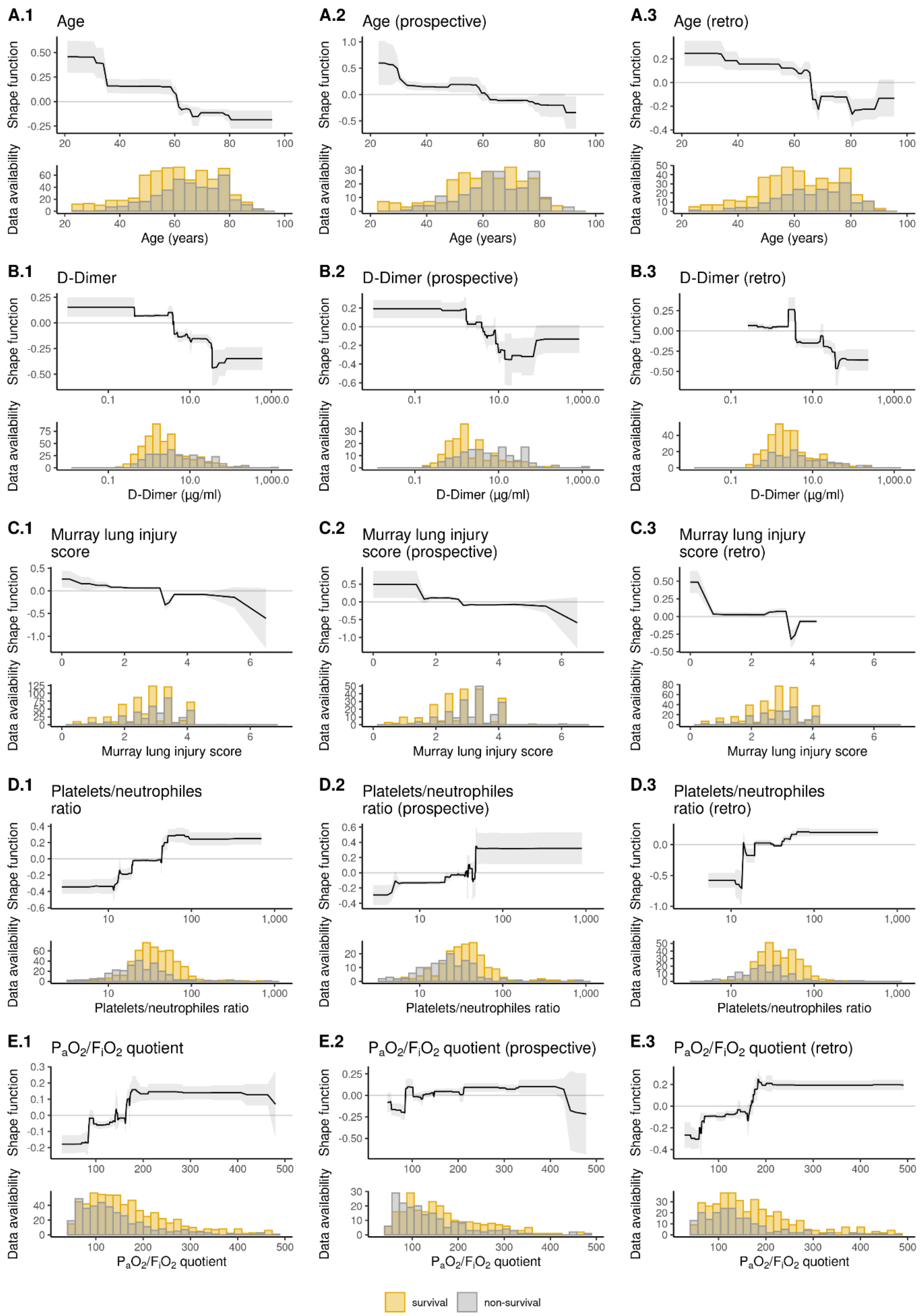

b) EBM model (10-interactions) for prediction of ECMO therapy

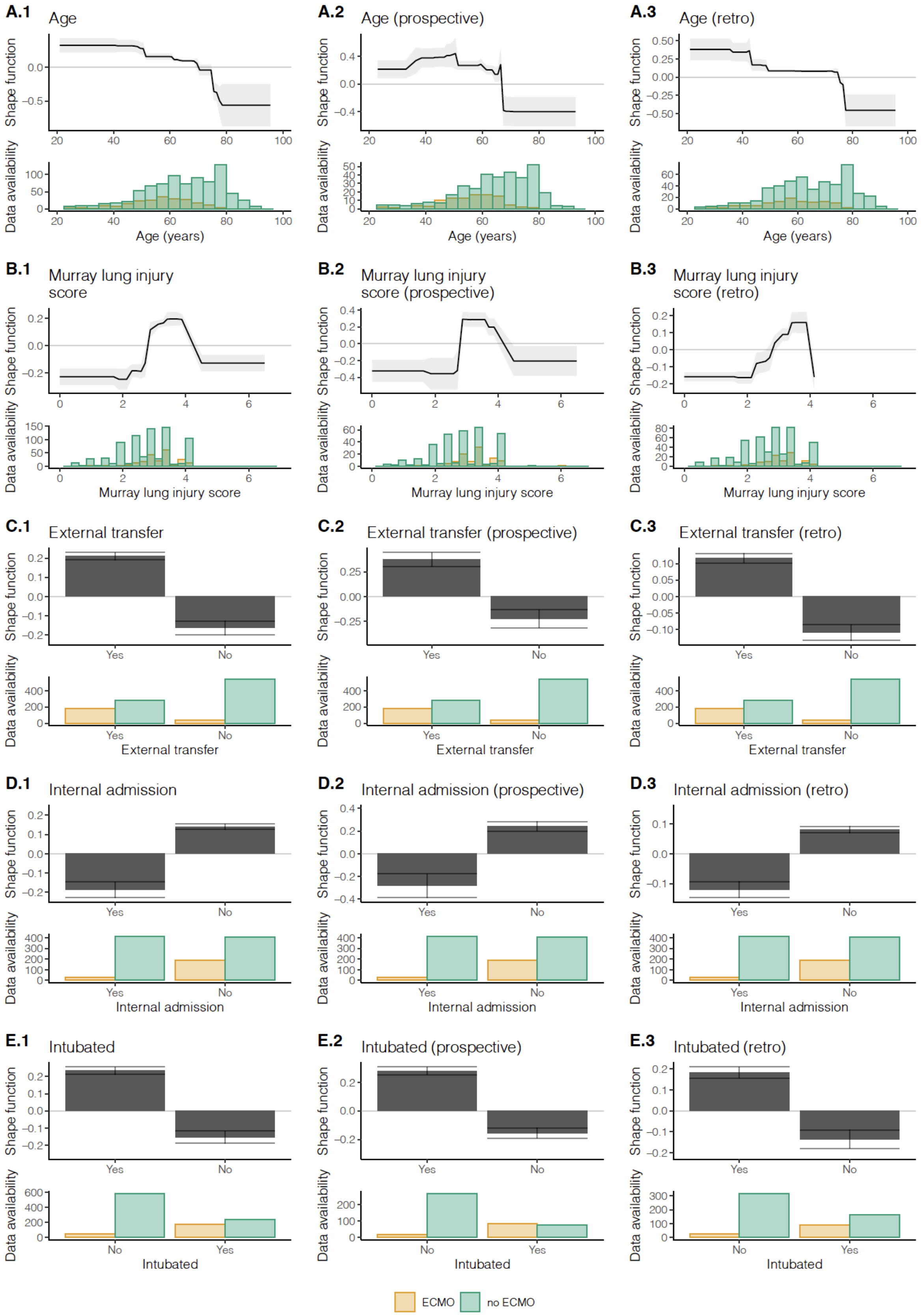

c) EBM model (10-interactions) for prediction of renal replacement therapy during ICU stay

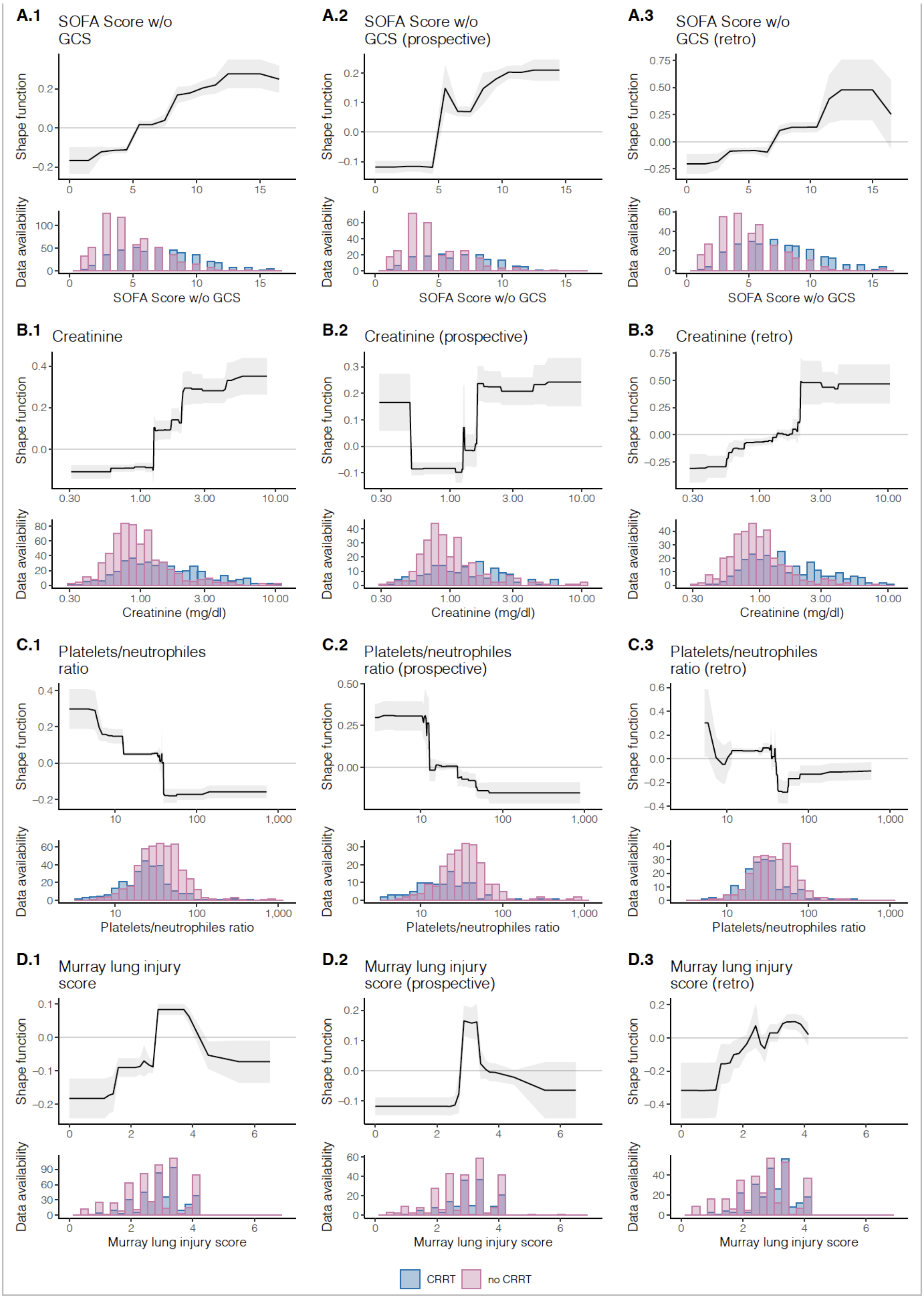

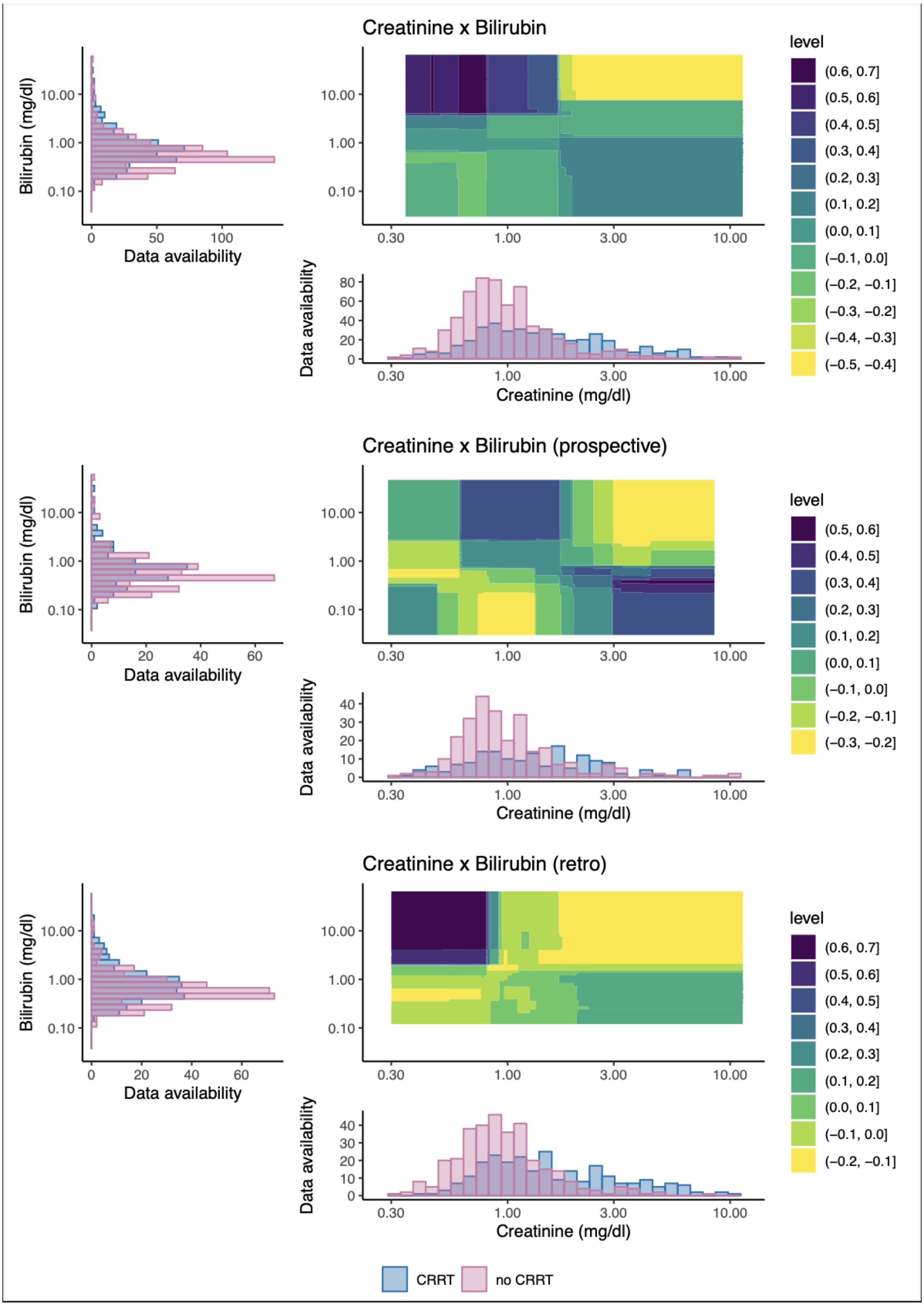
Creatinine x Bilirubin
Bilirubin (mg/dl)
Data availability
Creatinine (mg/dl)
level
(0.6, 0.7]
(0.5, 0.6]
(0.4, 0.5]
(0.3, 0.4]
(0.2, 0.3]
(0.1, 0.2]
(0.0, 0.1]
(−0.1, 0.0]
(−0.2, −0.1]
(−0.3, −0.2]
(−0.4, −0.3]
(−0.5, −0.4]
Creatinine x Bilirubin (prospective)
Creatinine x Bilirubin (retro)
CRRT
no CRRT

References

1. team Tpd: **pandas-dev/pandas: Pandas**. In.: Zenodo; 2020.
2. Pedregosa F, Varoquaux G, Gramfort A, Michel V, Thirion B, Grisel O, Blondel M, Prettenhofer P, Weiss R, Dubourg V *et al*: **Scikit-learn: Machine Learning in Python**. *J Mach Learn Res* 2011, **12**(null):2825–2830.
3. Caruana HNaSJaPKaR: **InterpretML: A Unified Framework for Machine Learning Interpretability**. In.; 2019.
